# Phytoplankton responses to repeated pulse perturbations imposed on a trend of increasing eutrophication

**DOI:** 10.1002/ece3.8675

**Published:** 2022-03-01

**Authors:** Julio A. A. Stelzer, Jorrit P. Mesman, Alena S. Gsell, Lisette N. de Senerpont Domis, Petra M. Visser, Rita Adrian, Bastiaan W. Ibelings

**Affiliations:** ^1^ Department F.‐A Forel for Environmental and Aquatic Sciences Institute for Environmental Sciences University of Geneva Geneva Switzerland; ^2^ Department of Ecosystem Research Leibniz‐Institute of Freshwater Ecology and Inland Fisheries Berlin Germany; ^3^ 9166 Department of Biology, Chemistry, and Pharmacy Freie Universität Berlin Berlin Germany; ^4^ Department of Aquatic Ecology Netherlands Institute of Ecology (NIOO‐KNAW) Wageningen The Netherlands; ^5^ Department of Ecology and Genetics Uppsala University Uppsala Sweden; ^6^ 1234 Department of Freshwater and Marine Ecology Institute for Biodiversity and Ecosystem Dynamics University of Amsterdam Amsterdam The Netherlands

**Keywords:** eutrophication, microbial ecology, preventive management, pulse perturbation, recovery, resilience

## Abstract

While eutrophication remains one of the main pressures acting on freshwater ecosystems, the prevalence of anthropogenic and nature‐induced stochastic pulse perturbations is predicted to increase due to climate change. Despite all our knowledge on the effects of eutrophication and stochastic events operating in isolation, we know little about how eutrophication may affect the response and recovery of aquatic ecosystems to pulse perturbations. There are multiple ways in which eutrophication and pulse perturbations may interact to induce potentially synergic changes in the system, for instance, by increasing the amount of nutrients released after a pulse perturbation. Here, we performed a controlled press and pulse perturbation experiment using mesocosms filled with natural lake water to address how eutrophication modulates the phytoplankton response to sequential mortality pulse perturbations; and what is the combined effect of press and pulse perturbations on the resistance and resilience of the phytoplankton community. Our experiment showed that eutrophication increased the *absolute* scale of the chlorophyll‐*a* response to pulse perturbations but did not change the *proportion* of the response relative to its pre‐event condition (resistance). Moreover, the capacity of the community to recover from pulse perturbations was significantly affected by the cumulative effect of sequential pulse perturbations but not by eutrophication itself. By the end of the experiment, some mesocosms could not recover from pulse perturbations, irrespective of the trophic state induced by the press perturbation. While not resisting or recovering any less from pulse perturbations, phytoplankton communities from eutrophying systems showed chlorophyll‐*a* levels much higher than non‐eutrophying ones. This implies that the higher absolute response to stochastic pulse perturbations in a eutrophying system may increase the already significant risks for water quality (e.g., algal blooms in drinking water supplies), even if the relative scale of the response to pulse perturbations between eutrophying and non‐eutrophying systems remains the same.

## Graphical abstract

Press (eutrophication) and repeated pulse perturbations are antagonistic forces shaping the microbial response to stochastic perturbations. Eutrophication increases the absolute response of the phytoplankton community to pulse perturbations, but does not change the proportionality of response compared to the pre‐event condition. Resistance increase, and recovery decrease after each pulse perturbation independently of the eutrophication process.

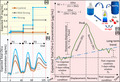

## 1 INTRODUCTION

Perturbations are an inherent phenomenon of any socio‐ecological system and can have far‐reaching consequences for the state of such systems. Perturbations can be pragmatically divided into press and pulse perturbations (Bender et al., 1984). Pulse perturbations are relatively instantaneous alteration of the physicochemical or biotic parts of the system that dissipates shortly after (e.g., storms), allowing the system to go back to its pre‐perturbation state if the main features of the system are preserved (e.g., species composition, habitat structure). This bouncing dynamic caused by a focal and transient perturbation is often referred as “*engineering resilience*” (*sensu* Pimm, 1984). Press perturbations, on the other hand, are sustained alteration that does not dissipate or leave the system (e.g., eutrophication), forcing the system to adapt in a way to accommodate this novel pressure, often by assuming a slightly different equilibrium. When the system fails in doing it so, the increase in environmental pressures forces the ecosystem toward an abrupt and persistent change in state over prolonged timescales. This dynamic of sustaining or shifting states is referred as “*ecological resilience*” (*sensu* Holling, 1973). Both antagonist frameworks of “*resilience*” may co‐exist into a press and pulse framework, potentially aiding our understanding of how complex systems respond to the interaction of multiple perturbations.

Aquatic systems sustain a multitude of fundamental ecosystem services that are vulnerable to perturbations. Drinking water supply, irrigation, and recreation are known to be temporarily disrupted by pulse perturbations of different natures as caused by extreme weather events (Khan et al., 2015; Ummenhofer & Meehl, 2017; WHO, 2011), waterborne diseases (Cann et al., 2013), and chemical spills (Anenberg & Kalman, 2019; Sengul et al., 2012). Although the most variate stochastic events have always impacted ecosystems, the frequency, intensity, and duration of weather‐related pulse perturbations are predicted to increase in a changing world (Bell et al., 2018; Harris et al., 2018; Woolway et al., 2021). Alongside, despite efforts to control eutrophication – one of the main freshwater press perturbations deteriorating water quality, its impact on ecosystems is also expected to rise due to climate change (Carr & Neary, 2008; Fink et al., 2018; Ho et al., 2019; World Water Assessment Programme, 2009). Eutrophication may enhance harmful cyanobacterial blooms (Huisman et al., 2018; Paerl & Huisman, 2009), alter long‐term ecological stability (Chapin et al., 2000; Rosset et al., 2014), and modify the structure of food webs (e.g., Alexander et al., 2017; van der Lee et al., 2021) resulting in substantial ecological, social, and economic losses (Dodds et al., 2009; Hoagland et al., 2002). With the aforementioned escalation in eutrophication combined with more prevalent stochastic pulse perturbations, water security is becoming an increasing concern as recognized by the United Nations’ sustainable development goals (UN, 2020).

There are multiple ways in which pulse perturbations and eutrophication as a press perturbation may interact. For instance, pulse perturbations may enforce stochastic mortality events, which initially reduces populational densities but also enhances dissolved nutrient concentrations due to cell lysis and increased turnover, promoting strong subsequent growth of primary producers (Haddad et al., 2008; Jacquet & Altermatt, 2020). When coupled with ongoing eutrophication, the amount of dissolved nutrients in the ecosystem that can be incorporated by primary producers to fix biomass increases, possibly increasing the peak response of the phytoplankton community in response to the combined effect of press and pulse perturbations.

While pulse perturbations trigger transient events, their consequences may become permanent at the ecosystem level (Harris et al., 2018; Scheffer et al., 2001). The distinction between transient and permanent responses to pulse perturbation is dependent on the capability of the system to recover from disturbance, which is often associated with (i) the compositional and functional structure of the ecosystem (Hillebrand & Kunze, 2020; Thayne et al., 2021), (ii) the legacy effect from repeated perturbations (Johnstone et al., 2016; Ryo et al., 2019), as well as with (iii) the rates of energy flow to higher trophic levels (McCauley et al., 2018; Shade, Read, et al., 2012). Eutrophication may modify these aspects by inducing changes in community composition (Rigosi et al., 2014; Rosset et al., 2014) and functional structure (Jochimsen et al., 2013; van der Lee et al., 2021; Moody & Wilkinson, 2019), which in the long run may have important implications for how an ecosystem responds to pulse perturbations. Many studies have posed that the long‐term changes in community‐level responses such as changes in species richness, composition, and/or dominance, directly or indirectly caused by press perturbations like eutrophication, can potentially be amplified by the effect of extreme weather events (Arens & West, 2016; Bertani et al., 2015; Boucek & Rehage, 2014; Smale & Wernberg, 2013), sometimes with catastrophic consequences for the conservation of ecosystems (Coumou & Rahmstorf, 2012; Harris et al., 2018).

Despite all our knowledge on the effects of freshwater eutrophication and stochastic events operating in isolation, we know little about how eutrophication as a press perturbation affects the response and recovery of aquatic ecosystems to pulse perturbations. Stochastic pulse perturbations are often short‐lived and challenging to observe in high resolution, unlike press perturbations that are persistent over time and practical to assess as part of long‐term monitoring campaigns (Stelzer et al., 2021). This hinders the simultaneous data collection of both types of perturbation, which is, however, needed to gauge how pulse and press perturbations may interact. Experimental approaches capable of mimicking long‐term press effects upon which stochastic perturbations are superimposed are a fundamental tool to increase our understanding of the topic (Yang et al., 2017). Moreover, comparatively little attention has been given to two other important aspects of perturbations. First, eutrophication is a dynamic process where pressure levels change over time, which is often neglected in laboratory and field experiments (Shade, Peter, et al., 2012; Stelzer et al., 2021). Second, repeated pulse perturbations produce a legacy effect carried over time in ecological communities (Jacquet & Altermatt, 2020; Johnstone et al., 2016), creating fundamentally different responses when compared to a single perturbation response (Ryo et al., 2019).

To help filling the knowledge gap on how continuous eutrophication interacts with sequential pulse perturbations, we performed a controlled press and pulse perturbation experiment using mesocosms filled with natural lake water of a meso‐oligotrophic lake. We started the experiment with three treatments under the same trophic state and thereafter applied two gradients of phosphate enrichment to simulate press perturbations of different rates, and a control. Together, we applied three sequential H_2_O_2_ shock treatments of variable intensities to induce mortality events with consequent nutrient release, mimicking the effect of pulse perturbations. With this experimental setup, we aimed to answer the following questions: (i) how does eutrophication modulate the phytoplankton response to sequential pulse perturbations? and (ii) how does eutrophication affect resistance and recovery of chlorophyll‐*a* (Chl‐*a*) levels after recurrent pulse perturbations?

## 2 MATERIALS AND METHODS

### 2.1 Field collection, acclimation, and build‐up of the mesocosms

Lake water was collected at the southernmost basin of Maarsseveense Plassen, Maarssen, The Netherlands (52°08′28.0″N, 5°04′59.9″E). Maarsseveense Plassen is a meso‐oligotrophic lake system with a surface area of 100 ha and a maximum depth of 34 m (see Swain et al., 1987 for an extensive description). Sampling consisted of taking 450 L of lake water, approximately 10 m from the shoreline and 30–70 cm below the surface (local depth 1.3–1.6 m). A single water sample was taken on 29th March 2019 and immediately filtered over a 2 mm mesh to remove most of the mesozooplankton.

In the laboratory, the 450 L tank was left standing for 10 min and had the bottom layer siphoned out to remove sand and other large particles. Next, the tank was homogenized and split into two batches. One batch was used for the experiment itself, while the other batch was retained and used to compensate sampling losses during the experiment (hereafter, refill water). The refill water was treated with a single pulse of 10 mg/L H_2_O_2_ (EMSURE^®^ Supelco^®^, Merck) and kept in the dark at 4°C, both to reduce biological activity until use in the experiment. Compensating sampling losses was needed due to the frequency and volume sampled (see Section 2.5). The refill water was chosen over other solutions to conserve the nutrient stoichiometry of the controls as similar as possible to their initial conditions.

The experimental batch was slowly acclimated to the experimental conditions for 24 days to avoid a temperature shock in the community. The long acclimation period was set due to the differences in water temperature between Maarsseveense Plassen at the sampling day (12°C) and the proposed 20°C “summer temperature” used in the experiment. During this acclimation phase, the temperature was increased at a rate of 2.5 ^o^C per week until reaching 20°C, and the incident light was set to 20 µmol photons m^−2^ s^−1^ (measured immediately above the water surface) with a photoperiod of 16:8 h (Light: Dark) simulating the sunset: sunrise period at the time of the experiment. No significant changes in phytoplankton pigment composition and concentration were observed during the acclimation process (data not shown). Water was circulated from the bottom of the experimental batch three times a day for 15 min using an aquarium pump (≈600 L/h) to reduce sedimentation during the acclimation.

**FIGURE 1:**
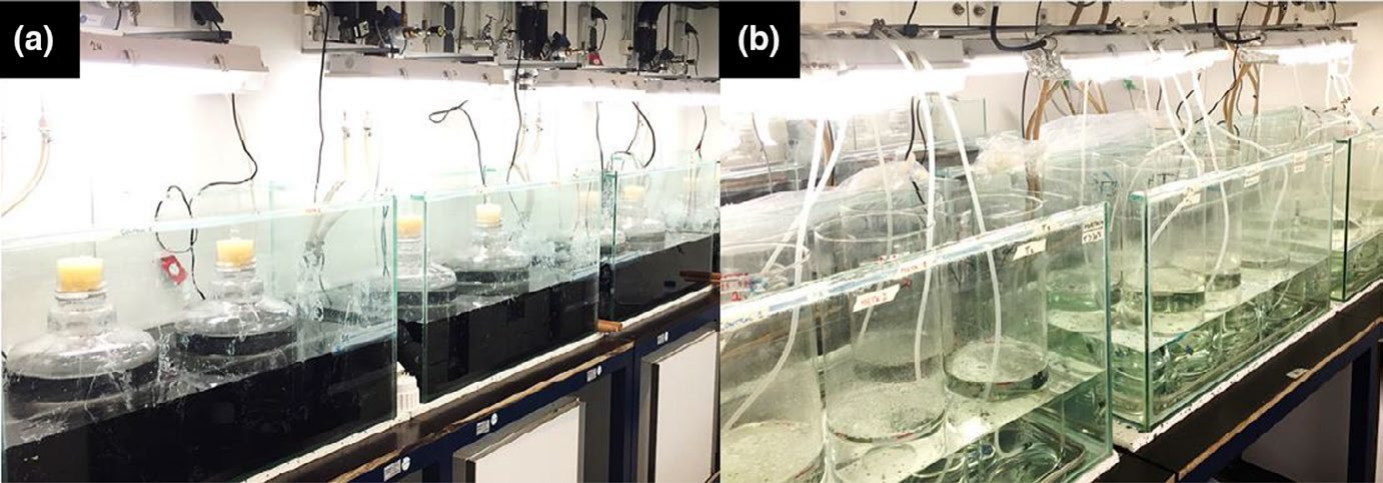
Experimental setup. (a) 10‐L carboys were filled with lake water and placed into large aquaria for light and temperature control. Light was accessible to the mesocosms through the top of the vessels, and the water around it was dyed in black to reduce the variability of response caused by diffusive scatter of light into the mesocosms. (b) Water vessels were used for H_2_O_2_ pulse perturbations. Water vessels stayed at the same temperature as the experiment and were aerated overnight (12–16 h) to keep particles resuspended

Three days before the beginning of the experiment, the experimental batch was homogenized, divided into eighteen 10‐L polycarbonate carboys (Nalgene, Rochester, New York, USA), and randomly placed inside large aquaria under identical light and temperature conditions as described above (Figure 1a). Each carboy received a non‐airtight rubber stopper to minimize aerial cross‐contamination and an electromagnetic stirring system. Stirring was set to turn on three times a day – 1 h in the morning during the sampling and 15 min in afternoon and night – with strength enough to resuspend sedimented particles without creating a vortex in the surface. To reduce light variability, the aquaria in which the carboys were submerged was dyed with “black pond dye” until light penetration became negligible (Secchi‐disk alike tool invisible at 5 cm below the surface, see Figure 1a).

### 2.2 Experimental design and application of the perturbations

The experimental design consisted of a control and two nutrient enrichment treatments (press perturbations) with six replicates each (*N* = 18). During the eutrophication process, mesocosms went through three cycles of pulse perturbations induced by a H_2_O_2_ shock treatment causing mortality events with consequent internal nutrient turnover. Pulse perturbations are fundamental for the quantifications of responses used here and therefore applied to all mesocosms (controls inclusive). The application of the press and pulse perturbations, sampling, and quantification of responses are described below. The experiment lasted 105 days, during which we quantified the effects of eutrophication on the community response to pulse perturbations.

#### 2.2.1 Press perturbations (eutrophication)

**FIGURE 2:**
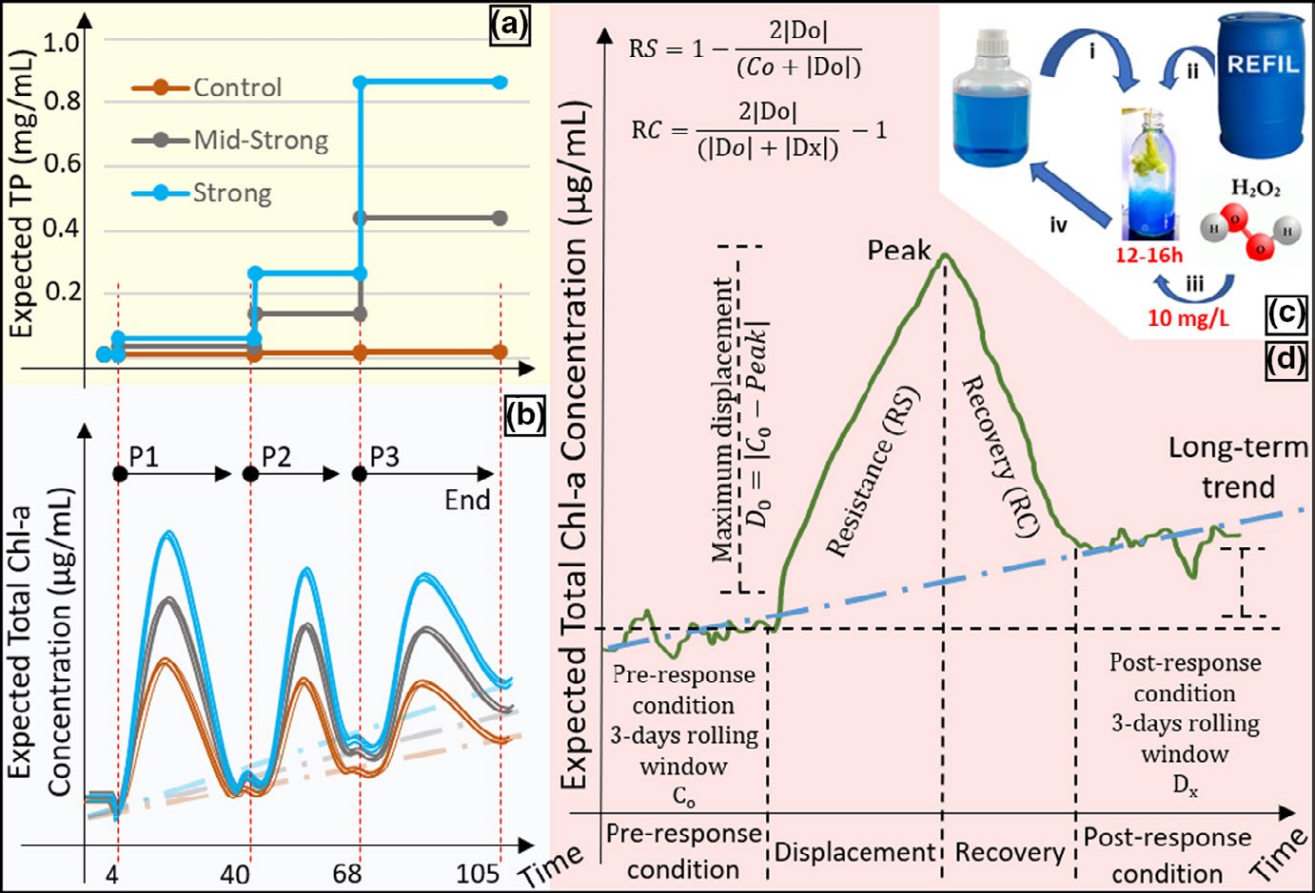
Infographic of the experimental design with its press and pulse perturbations, expected responses, and a conceptual example of how perturbation metrics were quantified. (a) The press perturbations consisted of a gradual increase in the phosphate concentration of the mesocosms, simulating the effect of eutrophication in three different treatments: (brown) control where no eutrophication took place; (gray) mid‐strong eutrophication with approximate final TP = 0.41 mg/L; and (blue) strong eutrophication with approximate final TP = 0.82 mg/L. The increase of the press perturbation happened in three stepwise increases during the experiment. (b) The three perturbation cycles with the expected responses of the mesocosms to the combination of the press (eutrophication) and pulse perturbations (H_2_O_2_ mortality events). The colored solid lines represent the expected development of Chl‐*a* during the experiment, each one with 6 replicates (*n* = 6, *N* = 18). The vertical red dotted line marks the exact moment of the pulse perturbation. P1, P2, and P3 were the three different perturbation cycles, each one with a different intensity and different eutrophication levels. The diagonal dashed lines indicate the expected long‐term changes in Chl‐*a* levels due to the combination of press and pulse perturbation (baseline changes). Pulse perturbations were simultaneous to the increase in the press perturbation. (c) Induction of the mortality pulse perturbations with consequent nutrient turnover. First, a fraction of the mesocosms were transferred to a separate container (i). To compensate for sampling losses, refill water was added to the same container where the pulse perturbation took place (ii). The sum of these two fractions was spiked with 10 mg/L H_2_O_2_ overnight (iii) before returning to the original mesocosms (iv). The fraction of the mesocosms split for the perturbation determined the intensity of the perturbations, P1 = 50%, P2 = 15%, and P3 = 30%. (d) Conceptual example of how perturbation metrics were quantified. Within an individual perturbation, the timeseries of the Chl‐*a* response to the pulse perturbation were divided into 4 moments (*x*‐axis). Resistance (RS), recovery (RC), and maximum displacement (Do) were individually calculated accordingly for each of the six replicates. The quantification of these metrics was used to model the effect of eutrophication (press perturbation) on the phytoplankton response to pulse perturbations. The green line represents the expected response of the system to the pulse perturbation (Time = 0). The blue dashed line indicates the long‐term changes in Chl‐*a* levels due to the combination of press and pulse perturbation (baseline changes)

**FIGURE 3:**
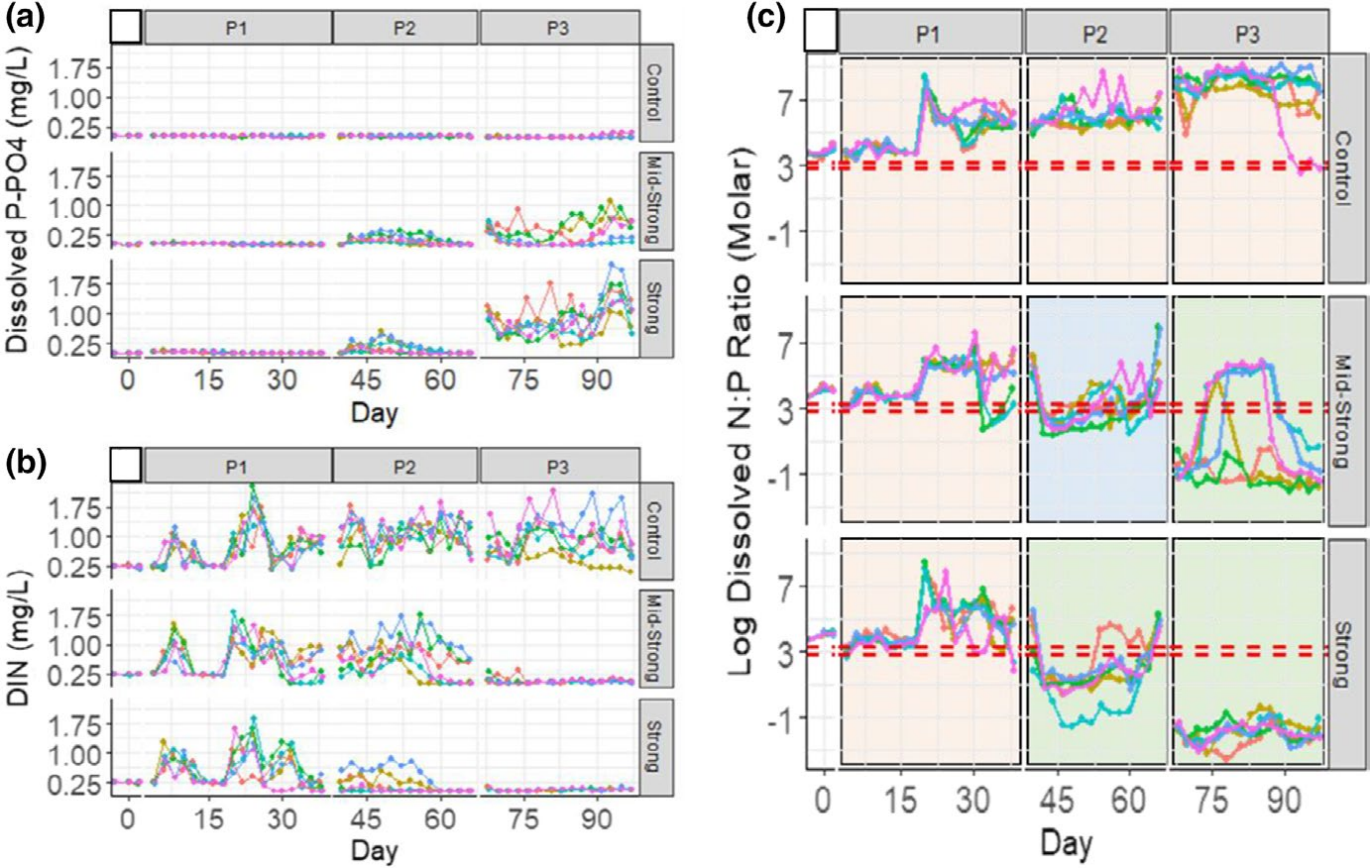
Timeseries of (a) dissolved orthophosphate; (b) dissolved inorganic nitrogen (sum of NH_4_, NO_2_, and NO_3_); (c) N:P ratio of dissolved nutrients during the three perturbation cycles of the experiment (P1, P2, and P3). Each colored line represents the temporal development of an individual replicate (*n* = 6, *N* = 18). Control, mid‐strong, and strong are the three eutrophication treatments (press perturbations). On panel c, the horizontal dashed red lines represent the N:P ratio of 16:1 (Redfield, 1934) and 25:1 (Sterner, 2009), and the shaded panels are an empirical indication of expected P‐limited systems (in orange, when the system is set above the horizontal lines), co‐limitation (in blue, when the system is set between the lines), and N‐limitation (in green, when the system is set below the horizontal lines)

At the beginning of the experiment, all the treatments started with the same total phosphorous concentration (TP ≈ 11.9 ± 5.3 µg/L). The control treatment remained at TP levels similar to the initial concentrations in the lake throughout the experiment, while the eutrophication treatments were exposed to three stepwise increases in TP (Figure 2a) by adding dissolved K_2_HPO_4_ (CAS‐16788‐57‐1; Merck) just after inducing the pulse perturbations. One of the treatments suffered a mid‐strong eutrophication process with predicted final TP = 0.41 mg/L, while the other treatment suffered a strong eutrophication process with predicted final TP = 0.82 mg/L (Figure 2a). The eutrophication treatments were set in a way that the mid‐strong eutrophication treatment would go through a full period of Nitrogen:Phosphorous (N:P) co‐limitation at the second perturbation cycle, while the strong treatment would abruptly shift from a P‐limited to an N‐limited system (see Figure 3c for reference). The amount of K_2_HPO_4_ added in each perturbation cycle to reach the next anticipated nutrient level was calculated using the dilution Equation (1), based on TP values measured on the days preceding the beginning of the first perturbation cycle.

Equation (1). Dilution equation for multiple solutions, where the final concentration in the mesocosms is a function of the partial dilutions divided by the total volume of the mesocosms. *C*_c_ is the predicted TP concentration in the mesocosms at the perturbation *p*; *V_c_* is the volume left in the mesocosms after sampling; *C_r_* is the TP concentration of the refill water; *V_r_* is the volume of refill water used; *C_s_* is the concentration of eutrophication solution; *V_s_* is the volume of eutrophication solution added and *V_c_* + *V_r_* + *V_s_* = 10 L.

(1) $$c_{c_{p}}=\left(c_{c_{p‐1}}v_{c_{p}}+c_{r_{p}}v_{r_{p}}+c_{s}v_{s_{p}}\right)÷\left(v_{c_{p}}+v_{r_{p}}+v_{s_{p}}\right)$$

#### 2.2.2 Pulse perturbations

Pulse perturbations were simultaneously applied to all the mesocosms (controls inclusive) at days 4, 40, and 68, constituting three perturbation cycles (P1, P2, and P3, respectively, Figure 2b). For this, mesocosms were homogenized, and a fraction of their volume was transferred to individual water vessels (Figures 1b and 2c_i_, *r* = 10 cm, *h* = 60 cm) where the H_2_O_2_ pulse took place. Additional to the mesocosms fraction, refill water was used to compensate for the volume lost due to sampling during the perturbation cycle (approximately 70 ml every other day, Figure 2c_ii_). Refill water has the same physicochemical composition as the control and therefore is used to conserve nutrient stoichiometry in the mesocosms, reducing nutrient losses caused by sampling. The sum of these two volumes constituted the proportion of the mesocosms subjected to the perturbation. Each perturbation cycle had a different volume fraction split for perturbation – P_1_ = 50%, P_2_ = 15%, and P_3_ = 30% – mimicking mortality events of different intensities (e.g., mortality induced to 50% of the community). The different perturbation intensities were induced as random effects in the system and not as a factorial design together with the eutrophication treatments. Hence, all the mesocosms received the same perturbation intensity at the same cycle.

The pulse perturbations themselves consisted of a single pulse of H_2_O_2_, acting as a shock treatment capable of bringing the system out of equilibrium. H_2_O_2_ leads to severe oxidative stress, inducing mortality with a consequent nutrient release when in high concentrations. Concentrations between 0.5 and 5 mg/L are reported to be somewhat selective, affecting cyanobacteria preferentially (Drábková et al., 2007). However, concentrations over this threshold are expected to induce mortality to most biological groups evenly (see Matthijs et al., 2012; Piel et al., 2021). Here, we spiked the content of the water vessels with 10 mg/L H_2_O_2_ (Hydrogen peroxide 30%, EMSURE^®^, Supelco^®^, Merck, Darmstadt, Germany, Figure 2c_iii_) overnight under similar conditions as in the carboys (light and temperature), except for the constant aeration used to keep particles in suspension (Figure 1b). The next day, residual H_2_O_2_ was measured in the water vessels using peroxide test strips Dosatest^®^ (VWR International, Cat no. 85433.601) before returning the perturbed fraction to the original mesocosms (Figure 2c_iv_). Residual H_2_O_2_ in the water vessel was never higher than 2 mg/L and virtually absent in the mesocosms within 24 h after completing the pulse perturbation. Since H_2_O_2_ is a biogenic reactive oxygen species (ROS) that decays to water and oxygen in the order of a few hours to a few days (Cooper & Zepp, 1990; Häkkinen et al., 2004), it leaves no persistent chemical footprint in the system. After completing the pulse perturbation, any deviation from the 10‐L mark in the mesocosms after returning the perturbed fraction was corrected using demineralized water to compensate for evaporation losses (mostly negligible during the experiment).

### 2.3 Sampling and data processing

Sampling was conducted every other day in the morning, one hour after the lights reached their full emission strength. Mesocosms were stirred for at least 15 min to allow for homogenization and particle resuspension (see Field collection, acclimation, and setup), followed by three counter‐vortex movements to reduce the effect of particle size separation due to fluid drag force before sampling. A sample of 250 ml was taken from the mesocosms and transferred to multiple conical polypropylene centrifuge tubes (VWR International) for further analyses (70 ml in total). The unused sampled volumes were immediately returned to the mesocosms, reducing sampling losses as much as possible.

#### 2.3.1 Pigment‐based community dynamics

Subsamples for pigment‐based community dynamics were kept in amber tubes, and their fluorescence was measured immediately after sampling using Phyto‐PAM (Walz, Germany). Phyto‐PAM estimates the relative abundances of different phytoplankton groups, i.e., cyanobacteria, green algae, and diatoms, based on the relative proportion of group‐specific pigments present in the sample (Walz, 2003). The conversion of relative abundances to absolute chlorophyll‐*a* concentrations (µg/L) was calculated based on pre‐determined calibration curves. We used *Microcystis aeruginosa*, *Chlorella* sp., and *Synedra* sp. cultures to generate the calibration curves for cyanobacteria, green algae, and diatoms, respectively. The total chlorophyll‐*a* concentration was calculated as the sum of the three chlorophyll‐*a* fractions. Data collection for pigment‐based community dynamics was done every other day over the full length of the experiment.

#### 2.3.2 Nutrients

Subsamples for dissolved nutrient analysis were immediately placed in the fridge (4°C) until concluding the fluorescence measurements on the same day. Next, 42 ml of sample were filtered through a 0.7 µm GF/F filter (Whatman, UK) mounted on a vacuum filtration manifold system. The filtrate was frozen at −20°C for later quantification of dissolved nutrients (P‐PO4, N‐NH4, N‐NO2, and N‐NO3) using an autoanalyzer (QuAAtro39 Autoanalyzer, Seal, USA).

#### 2.3.3 Size‐based community dynamics

Community size‐based biovolume distribution was assessed in every other sample (four days) using a Coulter Counter (Beckmann, Indianapolis – USA). Samples were pre‐filtered with a 100 µm‐mesh to avoid clogging, and particles with diameters between 2.93 and 60 µm were counted. The lower cut‐off 2.93 µm was set to avoid counting bacterioplankton and cell debris, while the upper cut‐off 60 µm was determined based on the abundance of the microbial community under microscopy. Particle surface and volume were quantified and allocated to 300 size bins. Three counts of 100 µl were averaged for each sample, and the total biovolume was calculated by summing the biovolume of all individual bins within a sample. Only a few missing cyclopods, filamentous algae, and one taxon of ciliates, all with negligible biovolumes compared to the range selected, were found after a taxonomic investigation under the microscope (Appendix S1: Functional structure). The total biovolume does not distinguish autotrophs from heterotrophs, thus, representing the dynamics of the whole microbial community.

### 2.4 Quantification of responses

#### 2.4.1 Long‐term effect of the press perturbation (eutrophication) on total biovolume and total chlorophyll‐*a* concentrations

To assess the effect of eutrophication on the biovolume build‐up of the <60 µm microbial community fraction, total biovolume was averaged during the whole perturbation cycle (from one H_2_O_2_ to the next H_2_O_2_ manipulation for the Baseline, P1, P2, and P3, Figure 2b). This was done for each replicate individually, totaling 72 averaged values (3 treatments × 6 replicates × 4 periods – including the pristine period). Therefore, each averaged value comprised the full biovolume of the microbial response to the combined effect of press and pulse perturbation, allowing a direct comparison between treatments. The relationship between biovolume build‐up and total chlorophyll‐*a* concentration was assessed using all data points available in both timeseries for each eutrophication treatment (*N* = 466).

#### 2.4.2 Short‐term effects of the press perturbation (eutrophication) on the response and recovery from mortality pulse perturbations (chlorophyll‐*a* as a proxy)

The dynamics of the response metrics preceding and following pulse perturbations were calculated based on three periods: (i) the pre‐response condition, (ii) the peak of the response, and (iii) the post‐event condition (Figure 2d). Using these three periods, we calculated the maximum displacement, resistance, and recovery of total chlorophyll‐*a* concentration for each one of the perturbations. We quantified 54 individual perturbations divided into three eutrophication treatments with six replicates, each one undergoing three perturbation cycles.

##### Pre‐response condition (*C*_o_) and post‐event condition (*D_x_*)

The determination of the pre‐response condition was automated using a minimum of 3 days moving average (2 or more sampling points), identifying the period of least variance within the perturbation cycle. This approach could identify the period just before the response to perturbation became apparent. Thus, the pre‐response condition does not necessarily represent the exact period preceding the H_2_O_2_ perturbation but the moment just before the system starts reacting to it. This approach is consistent with what often happens in real‐world systems since the response to pulse perturbations often shows a lag‐effect (Dodson et al., 2000) – a difference in timing between the perturbation and the response. Furthermore, this approach allows uniformity when assessing sequential perturbations (as in this experiment), where the pre‐response condition of a given perturbation cycle is also the post‐event condition of the perturbation cycle just before it (e.g., P2_Post‐event condition_ = P3_Pre‐response condition_).

##### Maximum displacement (*D*_o_)

The maximum displacement was calculated as the absolute difference in units of chlorophyll‐*a* between the peak of the response (maximum Chl‐*a* value at the perturbation cycle) and the pre‐response condition (*C*_o_) (Equation 2). The maximum displacement shows the *absolute* effect of the pulse perturbation given their respective eutrophication treatments rather than the *relative* effect (see resistance and recovery index). Thus, it represents the magnitude of the response to the pulse perturbation, irrespective of the Chl‐*a* level before the response starts.

Equation (2). Calculation of maximum displacement.

(2) $$D_{0}=\left|C_{0}‐\text{Peak}\right|$$

##### Resistance (RS) and Recovery index (RC)

Resistance and recovery index were calculated following the method proposed by Orwin and Wardle (2004) with slight modifications. Both indexes are based on percent changes to the pre‐response condition, holding proportionality between treatments of different trophic states. Therefore, the RS and RC allow the observation of how eutrophication modifies the intensity of response to the pulse perturbation, being a relative index that allows direct comparison between systems with different chlorophyll‐*a* levels.

**FIGURE 4:**
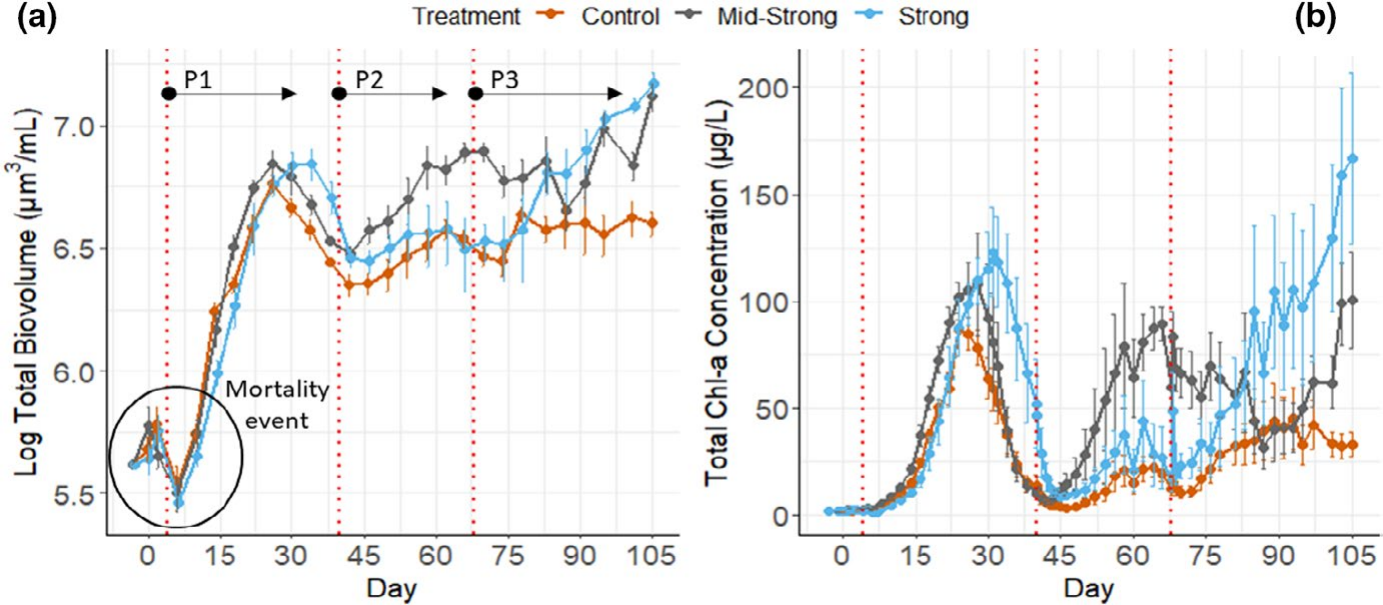
The microbial community responses to the combination of press and pulse perturbations along the 105 days of the experiment. (a) Timeseries of total biovolume of the whole microbial community (2.93 µm < fraction <60 µm) and (b) timeseries of total chlorophyll‐*a* concentration, a proxy for the response of the phytoplankton community. Control, mid‐strong, and strong are the three different levels of continuous eutrophication (press perturbation). Vertical dashed lines represent the exact moment when the mortality pulse perturbations were induced to the system; P1, P2, and P3 are the different pulse perturbation cycles. Solid lines represent the mean value of the response variable (*n* = 6), and error bars are the mean ±1 standard error of the mean (SEM)

The resistance index (RS) represents the degree of change the pulse perturbation caused in the system compared to the undisturbed situation (Equation 3) – originally proposed as an undisturbed treatment and here proposed as the pre‐response condition within the perturbation cycle:

Equation (3). Calculation of the resistance index.

(3) $${\text{RS}}_{\left(p\right)}=1‐\frac{2\left|D_{0}\right|}{\left(C_{0}+\left|D_{0}\right|\right)}$$

where RS_(p)_ is the resistance at the perturbation cycle “*p*”; *D*_o_ is the difference between the pre‐response condition (*C*_o_) and the peak of disturbance (see Figure 2d). The behavior of the index can be visualized in the Figure S1. The original index is restricted to values between −1 and +1, with the value of +1 showing that the perturbation had no effect on the system (maximal resistance). Lower values indicate stronger effects of the perturbation, with RS = 0 representing 100% change compared to the pre‐response condition (e.g., *C*_o_ = 20, *D*_o_ = 20, RS = 0; 100% change compared to the pre‐response condition level). The index scales change exponentially, and negative values indicate that the system was displaced by more than 100% compared to the pre‐response condition. To expand the index within the values of low resistance (values between 0 and −1), we rescaled it to values between 0 and 10, where 10 represents no change (original index = 1), 9 represents 100% change (original index = 0) compared to the pre‐response condition, and smaller values represent less resistance. The formulas used for rescaling are provided as Table S1, as well as the comparison between original and rescaled indexes (Figures 2 and 4).

The recovery index (RC) is based on the concept of engineering resilience as proposed by Pimm (1984), in which the system has a “single” equilibrium to which it may return after dissipating the pulse perturbation. Yet, once the perturbations are followed by changes in trophic state, we expect the post‐event conditions to stabilize at a higher chlorophyll‐*a* concentration than the pre‐response condition – mainly because of the increased carrying capacity caused by the nutrient addition. The recovery index incorporates this situation into its calculation and determines to what extent the system recovers after perturbation (fully or partially), considering the pre‐response condition, the post‐event condition, and the maximum displacement (Equation 4).

Equation (4). Calculation of the recovery index.

(4) $${\text{RC}}_{\left(p\right)}=\frac{2\left|D_{0}\right|}{\left(\left|D_{0}\right|+\left|D_{x}\right|\right)}‐1$$

where RC_(p)_ is the recovery at the end of perturbation cycle “p” and *D_x_* is the post‐event condition of the perturbation cycle (Figure 2d). Similar to resistance, recovery is also originally restricted to values between −1 and +1, with the value of +1 showing that the system fully recovered after the perturbation (maximal recovery). Lower values indicate loss of recovery, with RC = 0 representing 0% recovery compared to the peak of the perturbation (e.g., *C*_o _= 10, Peak = 20, *D_x_* = 20, RS = 0; 0% recovery compared to the peak of perturbation). As RS, the RC scale changes exponentially as further it goes from +1 (see Figure S3). Also, RC uses the maximum displacement as a scaling factor to calculate recovery to the pre‐response condition. To expand the index within the values of high recovery (values between 1 and 0), we also rescaled it to values between 0 and 10, where 10 represents *full recovery* (original index = 1), 1 represents *no recovery* (original index = 0) compared to the pre‐response condition, and values smaller than 1 mean that the system has drifted away after the perturbation (see Table S1 for rescaling).

### 2.5 Statistics

All statistics were calculated using the R software (R Core Team, 2020). Pre‐response condition, maximum displacement, and recovery index were compared between treatments and perturbation cycles by linear mixed effect models (LMEM) using the package “lme4” (Bates et al., 2015). Resistance index and total biovolume were assessed in the same way by generalized additive mixed model (GAMM) using the package “mgcv” (Wood, 2017). LMEM and GAMM were set to calculate effect size estimates of the treatments compared to the control. Thus, significance and estimates were calculated using the control as a reference. Because the control treatment does not suffer eutrophication, temporal changes in its response to pulse perturbations are assumed to be a consequence of the sequential pulse perturbations; while its differences to the eutrophying treatments, the effect of eutrophication. All the models had the number of pulse perturbations and eutrophication treatment as fixed effect terms, tested with and without interactions. The intensity of pulse perturbations (fraction of the mesocosms spared for the pulse perturbation) and temporal pseudo‐replication were set as random effect terms, with the second allowing for random slopes based on the perturbation effect when model complexity allowed (Appendix S1: Statistics). Results are expressed as a function of the number of sequential pulse perturbations since neither resistance, recovery, nor maximum displacement are time‐dependent metrics on the way they were calculated. The effect of total biovolume and eutrophication on total chlorophyll concentration was assessed by a generalized linear model (GLM), including an interaction between the terms to include potential changes in chlorophyll‐*a* concentrations per biovolume unit. GLM was fitted using the package “lattice” (Sarkar, 2008). To comply with the assumptions for model validation, data were log10‐transformed when needed. Significance values were obtained from *F*‐distributions, and *p*‐values <.05 are referred to as *significant*.

## 3 RESULTS

### 3.1 The overall response to pulse perturbations

The eutrophication treatments (press perturbations) successfully shifted the systems from a P‐limited system to an N‐limited system by the end of the experiment (Figure 3a/b). Also, we successfully created a period of likely N:P co‐limitation at the second perturbation cycle for the mid‐strong treatment (Figure 3c). The co‐limitation period represents a fundamental stoichiometric difference between the two eutrophication treatments. Yet, the co‐limitation period did not result in substantial differences in Chl‐*a* responses to pulse perturbations between them. Controls remained P‐limited during the whole experiment.

The H_2_O_2_ pulse perturbations induced mortality events with a consequent nutrient release, as observed by the abrupt drop in total biovolume (Figure 4a) and peaks of dissolved inorganic nitrogen (Figure 3b) that followed the perturbations. Shortly after the induced mortality and nutrient turnover, the phytoplankton community showed a positive growth response observed by the peaks in Chl‐*a* levels (Figure 4b). Also, the pulse perturbation increased the total biovolume of the microbial community (2.93 µm < fraction < 60 µm). The increase in total biovolume also happened in the controls, where no nutrients were added during the experiment (no press perturbation). The Chl‐*a* and total biovolume response were transient and followed a recovery period, which varied between treatments and perturbation cycles. The first pulse perturbation induced marked changes in the total biovolume of the microbial community, which contrary to Chl‐*a*, did not recover until the end of the experiment. The combination of eutrophication and pulse perturbations resulted in systems with different functional structures (Appendix S1: Functional structure), chlorophyll‐*a* levels, and biovolumes of the microbial community (Figure 4), which affected the pattern of response to pulse perturbation of the phytoplankton communities (Table 1). The periphyton formation was quantified in the last quarter of the experiment, with no major development observed in the data (Appendix S1: Periphyton formation).

**TABLE 1:** Statistical summary from the results of the perturbation metrics assessing the effect of eutrophication (press perturbation) on the Chl‐*a* response to a mortality pulse perturbations with internal nutrient turnover

Metric	Model	Press perturbation (eutrophication)	Pulse perturbation (mortality + turnover)	Interaction
Pre‐response	LMEM	n.s	***F* = 50.01, *p *< .001**	***F* = 4.58, *p *< .01** ^mid−strong^
Max displacement	LMEM	***F* = 6.15, *p *< .01**	n.s	n.a
Resistance	GAMM	n.s	***F* = 22.47, *p *< .01**	n.s
Recovery	LMEM	n.s	***F* = 17.16, *p *< .01**	n.s

Note

*F* and *p* are the *F*‐value and *p*‐value from the model output. The fields in bold are statistically significant.

Abbreviations: GAMM, generalized additive mixed model; LMEM, linearized mixed effect model; n.a, not assessed; n.s, non‐significant.

### 3.2 Long‐term effect of the press perturbation (eutrophication) on total biovolume and total chlorophyll‐*a* concentrations

**FIGURE 5:**
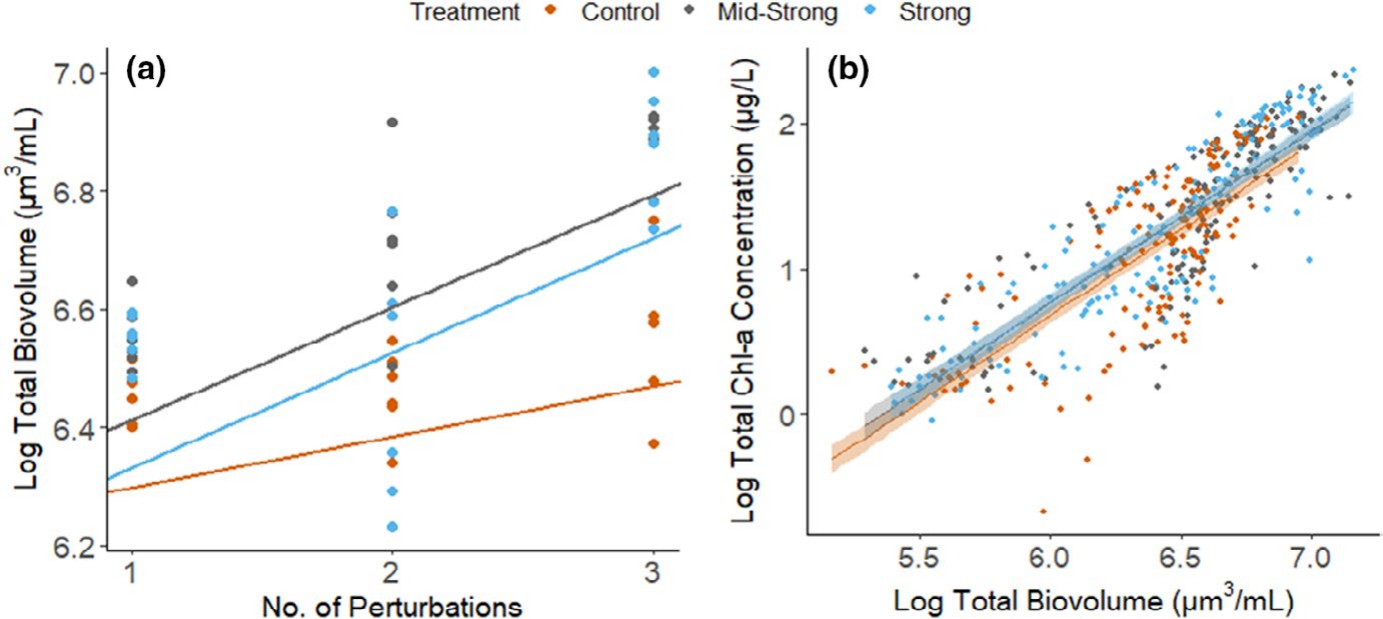
The effect of press and pulse perturbations on the total biovolume of the microbial community (2.93 < fraction <60 µm) and its correlation to Chl‐*a* levels. (a) The effect of eutrophication and pulse perturbations on the biovolume accumulation during the experiment. The solid regression lines represent the full linear mixed‐effect model estimates, and the dots are the empirical data (*n* = 6). (b) Generalized linear model showing the regression between total biovolume and total chlorophyll‐*a* for each one of the eutrophication treatments (press perturbation). The solid lines represent the model estimates and the shaded area the 95% confidence interval of the model. Dots are empirical data (*N* = 466, *R* ^2^ = .723)

Perturbations induced a significant increase in biovolume of the particle fractions smaller than 60 µm (*F* = 5.52, *p *< .05, larger fractions were not quantified). Also, eutrophication interacted with the perturbations (*F* = 3.30, *p *< .05), accelerating the rate of biovolume build‐up in the two nutrient‐rich treatments (Figure 5a). Biovolume showed to be positively correlated to the total chlorophyll‐*a* concentration in the mesocosms (*p *< .001, *R* ^2 ^= 0.723), suggesting a coupled increase of biovolume and primary producers (Figure 5b). The different eutrophication rates also changed the relation between biovolume and chlorophyll‐*a* concentration, meaning that chlorophyll‐*a* concentrations were higher in the eutrophied treatments than in the control treatment for the same given biovolume (*p *< .05). Yet, these differences were small (an increase of ≈ 1.2 µg/L of total chlorophyll‐*a* compared to the control).

### 3.3 Short‐term effects of the press perturbation (eutrophication) on the response and recovery from mortality pulse perturbations (chlorophyll‐*a* as a proxy)

#### 3.3.1 Pre‐response conditions

**FIGURE 6:**
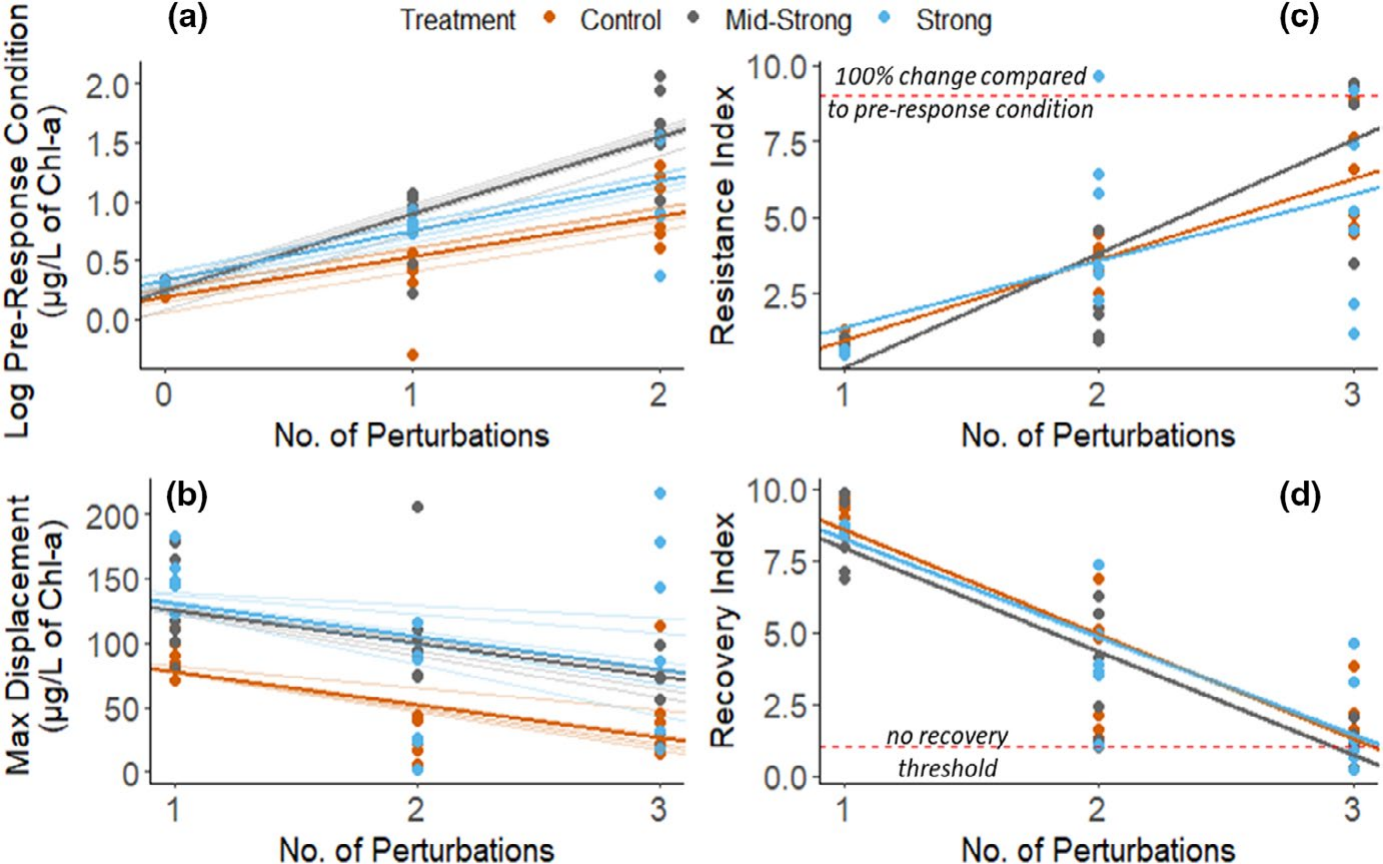
The effect of eutrophication on the Chl‐*a* response to the mortality pulse perturbations. Solid regression line represents the full model estimates, and shaded lines represent the individual mesocosms estimate with or without random intercepts depending on the model complexity. Dots represent the metrics of pulse perturbation calculated from the empirical data (*n* = 6). (a) Changes in the total chlorophyll‐*a* concentration at the pre‐response condition; (b) maximum displacement of total chlorophyll‐*a* concentration; (c) resistance Index, and (d) recovery Index

The total chlorophyll‐*a* concentration in the pre‐response conditions increased significantly during the experiment. From P1, P2 to P3, the Chl‐*a* concentration increased by a factor of 6 in the controls, by a factor 26 in the mid‐strong treatment, and by a factor of 10.5 in the strong eutrophication treatment (an absolute increase of 9.0, 52.2, and 18.1 µg Chl‐*a*/L, respectively). The sequential pulse perturbations showed a higher effect size for modifying total Chl‐*a* concentrations at the pre‐response condition than the eutrophication pressure itself (Appendix S1: Statistics). The increase in Chl‐*a* levels at the pre‐response conditions happened in all the treatments and was caused by the sequential pulse perturbations (*F* = 50.01, *p *< .001). Moreover, our assessment indicated that eutrophication interacted with the effect of perturbations (*F* = 4.58, *p *< .01), meaning that the rate of change in Chl‐*a* concentration due to the repeated perturbations was amplified by eutrophication (Figure 6a). Yet, we observed no isolated eutrophication effect in Chl‐*a* levels changes at the pre‐response conditions (*F* = 5.19, *p* < n.s). The random effect size estimates of the different intensities of pulse perturbations applied to the mesocosms in the previous perturbation cycle showed a minor effect on the chlorophyll‐*a* levels at the pre‐response condition (Appendix S1: Statistics). Eutrophication and the number of perturbations explained 67% of the variance in the model, while the full model explained 75% of the observed patterns in Chl‐*a* concentrations in the pre‐response conditions (marginal *R* ^2 ^= 0.676/conditional *R* ^2 ^= 0.751).

#### 3.3.2 Maximum displacement

The absolute increase in total Chl‐*a* concentration between the pre‐response condition and the peak of the response was higher in both eutrophication treatments compared to the controls (*F* = 6.15, *p *< .01). This means that eutrophication increased the magnitude on which the phytoplankton community responded to the pulse perturbations. Still, both eutrophication treatments responded similarly, irrespective of the nutrient co‐limitation that developed in the mid‐strong treatment. For both treatments, the absolute number of total chlorophyll‐*a* units (µg/L) displaced between the pre‐response condition and the peak was nearly twice as large as the control. Model estimates showed that the eutrophication treatment produced an effect size in the opposite direction of the number of perturbations (antagonistic response). While eutrophication showed a positive effect size on maximum displacement, serial perturbations resulted in a negative one. Yet, the sequential pulse perturbations hitting the system did not significantly affect the maximum displacement, despite the observed negative trend (*F* = 1.89, *p* < n.s) (Figure 6b). Part of this result is addressed by the strong random effect sizes of perturbation intensity, which corrects the fixed effect sizes of the model (Appendix S1: Statistics). A separate analysis of the effect of perturbation intensity on maximum displacement showed a significant increasing trend, indicating that indeed maximum displacement and perturbation intensity were positively correlated – and both negatively correlated to sequential perturbations (Appendix S1: Statistics). For this reason, the interaction between eutrophication levels and the number of perturbations was not tested. The model showed a full variance explained of 55% with eutrophication and the sequential perturbations explaining 28% of the observed variance.

#### 3.3.3 Resistance

Only six times out of 54 (11%), the mesocosms displaced less than double of the pre‐response condition (points above the red line, Figure 6c), indicating that the system was very susceptible to the pulse perturbations applied. The resistance significantly increased during the experiment mainly due to the impact of serial perturbations (*F* = 22.47, *p *< .01, *R* ^2 ^= 0.594). The nutrient enrichment treatments did not significantly affect the resistance level compared to the controls (*F* = 1.46, *p* < n.s), nor did eutrophication interact with the perturbations (*F* = 2.02, *p* < n.s). The resistance index is calculated as a relative change between the pre‐response condition and the conditions at the peak of the perturbation, therefore, normalizing the different scales of response caused by the press perturbation as observed in the maximum displacement (see Resistance (RS) and Recovery index (RC)).

#### 3.3.4 Recovery

Recovery of Chl‐*a* levels in the systems was significantly affected by the sequential perturbations (*F* = 17.16, *p *< .01), with some mesocosms losing the capability to recover within the timeframe of the experiment after the third perturbation cycle (Figure 6d). We found no statistically significant indication that eutrophication reduced or interacted with sequential perturbations to reduce recovery after the pulse perturbation (*F* = 0.90, *p* < n.s and *F* = 0.182, *p* < n.s; respectively). The full model explained 82% of the observed variance, with eutrophication and the number of perturbations accounting for 71% (marginal *R* ^2 ^= 0.708/conditional *R* ^2 ^= 0.822).

## 4 DISCUSSION

Here we explored the interplay between eutrophication and sequential pulse perturbations on the response of the phytoplankton community. Our results showed that resistance increased and recovery decreased after each pulse perturbation and that eutrophication per se can increase the magnitude of response of the phytoplankton community compared to non‐eutrophying systems. Yet, we did not find evidence that eutrophication would make the phytoplankton community proportionately less resistant or less resilient to pulse perturbations than when eutrophication is absent. Although experiments and field observation explicitly considering a press and pulse framework assessing the interactive effect of eutrophication on the phytoplankton response to pulse perturbations are virtually absent, similar results were observed on the effect of droughts in eutrophying grassland (Bharath et al., 2020; Xu et al., 2014).

The H_2_O_2_ pulse perturbations induced community‐level mortality events, creating a transient period of high autochthonous dissolved nutrient levels in the mesocosms. With dissolved nutrients available for uptake, transient peaks in chlorophyll‐*a* concentration were observed. This response pattern was already described in other microbial communities (Haddad et al., 2008; Jacquet & Altermatt, 2020) and advocated to describe phytoplankton responses to extreme weather events that induce mortality with consequent increases in nutrient availability and turnover. Moreover, it is similar to what is described in lakes after a storm, where phytoplankton is mixed in the water column reducing cell density; at the same time that prompts nutrient upwelling with subsequent opportunities to increase Chl‐*a* concentrations (Stockwell et al., 2020).

The first perturbation cycle showed the lowest resistance of the phytoplankton community, with systems reaching chlorophyll‐*a* levels about 40 to 60 times higher than the pre‐response condition. Such an intense response was unexpected and most likely associated with the novelty of the perturbation to the microbial community (Johnstone et al., 2016), which has never been exposed to H_2_O_2_. Another possible explanation is that the chemical effect of hydrogen peroxide – as a strong oxidizer capable of breaking down stable dissolved organic matter that was not promptly bioavailable before the first perturbation – turned over more nutrients at the first perturbation cycle, thereby increasing the phytoplankton response. Also, the first pulse perturbation had the highest intensity. Yet, our results did not point to a major contribution of perturbation intensity to the resistance of the phytoplankton community on the different perturbation cycles (Appendix S1: Statistics).

As presented before, pulse perturbations *per se* permanently increased the biovolume of the microbial community within 2.93–60 µm even when the press perturbation was absent. This suggests that the community structure was permanently changed despite the observed recovery in chlorophyll‐*a* (Appendix S1: Functional structure). Differences between compositional and functional recovery after pulse perturbations have been exhaustively reviewed and are well known to diverge in mesocosms experiments (Hillebrand & Kunze, 2020; Shade, Peter, et al., 2012), mainly due to the lack of dispersal and differences in seeding (Hillebrand & Kunze, 2020). In our mesocosms, the changes in community composition sustained the functional redundancy necessary to rebuild the Chl‐*a* levels. Similar results are broadly described in the literature with different systems and scales (Allison & Martiny, 2008; Allan et al., 2011; Hoover et al., 2014; Pennekamp et al., 2018). However, from the second perturbation onwards, the mesocosms partially lost their capability to fully recover within a perturbation cycle, irrespectively of the eutrophication level. These incomplete recovery patterns led to post‐response conditions stabilizing at a higher chlorophyll‐*a* concentration compared to the pre‐response condition, enforcing the formation of novel baselines along the experiment. At the third perturbation cycle, some mesocosms reached a point of no recovery (transformed RC_index_ = 1).

While pulse perturbations increased the amount of autochthonous nutrients in the mesocosms, eutrophication increased the amount of allochthonous nutrients. Still, despite similar patterns in nutrient availability, press and sequential pulse perturbations had different effects on the mesocosms. Mortality pulse perturbations are reported to reduce population sizes of species with lower‐intrinsic growth rates, selecting for species of high‐intrinsic growth rates that can rapidly recover from the pulse perturbation (Haddad et al., 2008). Thus, smaller primary producers known for their high intrinsic growth rates when nutrients are available (Ward et al., 2017) would dominate a perturbed system if top‐down regulation cannot be sustained at high levels – which would act more strongly on small than large‐sized phytoplankton. In the same response direction, eutrophication increases nutrient availability creating favorable conditions for fast‐growing taxa (Klappenbach et al., 2000). These long‐term interactions on nutrient availability between press and pulse perturbations potentially explain the biovolume build‐up within the 2.93–60 µm fraction and why the eutrophied treatments showed higher biovolumes with higher Chl‐*a* peaks in response to pulse perturbations. However, at the same time that pulse perturbations select for small primary producers, the sequential pulse perturbations may artificially select for organisms that cope better with the pulse perturbation regime, reducing the responsiveness of the system (but note that only a part of the community was subjected to pulse perturbations). Thus, the more pulse perturbations, the higher the resistance of the community, and the lower the observed response to the internal nutrient turnover. Hence, the effect of pulse perturbations may act in either way depending on the number of perturbations suffered by the systems. Moreover, the effect of sequential pulse perturbations and eutrophication can be interpreted as forces that act in the opposite direction when modulating the community response to a stochastic event that induces internal nutrient turnover. In our experiment, the build‐up of resistance due to sequential pulse perturbations was more significant than the combined effect of eutrophication and internal nutrient turnover caused by the pulse perturbations.

Our experimental mesocosms suggested that community structure was shaped to absorb the sequential pulse perturbations at the cost of increasing resistance and reducing recovery. The more perturbed the system was, the less responsive it became, and this can be interpreted as a possible community pathway toward stability (Paine et al., 1998). The loss of recovery and increase in resistance driven by the serial perturbations rather than eutrophication was evidenced in multiple aspects of the system. First, the controls showed a decrease in recovery and increase in resistance, despite not having any ongoing eutrophication process. Second, the eutrophying treatments did not show any difference compared to the control at any perturbation cycle, indicating that eutrophication had no observable effect in the loss of recovery or increase in resistance. Third, the effect sizes obtained from the LMEM and GAMM showed a prevalence of repeated perturbations over eutrophication (Figures S10 and S11) for determining recovery and resistance. This combination of evidence suggests that the sequential pulse perturbations can be more important than the trophic state for phytoplankton community stability. The recovery and resistance indices observed here were within the range of distribution observed in the shallow eutrophic Lake Müggelsee (Thayne et al., 2021) while studying the lake response to a multitude of storms across different seasons. There, the pre‐response conditions partially controlled the stability of the lake when hit by extreme storm events. However, press perturbations were not explicitly considered (i.e., changes in the lake's trophic state).

Moreover, we observed that microbial communities undergoing strong eutrophication are likely to show an increase in chlorophyll‐*a* levels after sequential pulse perturbations compared to non‐eutrophying systems (nearly twice as much). Since eutrophic systems often already show higher Chl‐*a* concentrations (Søndergaard et al., 2011), the combination with larger chlorophyll‐*a* displacements may dramatically increase the immediate consequences of the pulse perturbations. This is an important consideration when managing waterbodies with strict regulatory directives (e.g., strict Chl‐*a* concentration thresholds for water supply) because eutrophication will not only affect the functioning of the system under stable conditions (Alexander et al., 2017; Jeppesen et al., 2005) but may also compromise water security when affected by stochastic events.

We were able to assess three pulse perturbations, while the number of perturbations hitting an aquatic system within a legacy effect window is likely higher in natural systems (Johnstone et al., 2016). It is possible that the effect of eutrophication on resistance and recovery of sequential perturbations would become explicit when assessing more perturbation cycles. Moreover, dispersal and multitrophic levels of natural complexity could also alter the legacy effect carried over the perturbations through species reseeding (Hillebrand & Kunze, 2020) and/or a stronger top‐down control of the phytoplankton community (McCauley et al., 2018), potentially modifying the resistance and recovery index. These processes may create significant differences between the experimental results we observed and their direct applications to natural lakes. Nevertheless, the nature of the processes described here may remain valid despite the changes in their relative importance at a whole‐lake level and work as a guiding framework for future studies.

## 5 CONCLUSION

The press and pulse experimental framework proposed here indicated that eutrophication and sequential pulse perturbations are forces that potentially act in opposite directions, with a prevalent effect of sequential pulse perturbations in the recovery and resistance of the phytoplankton community. Eutrophication increased the *absolute* response (magnitude) of the phytoplankton community to pulse perturbations but did not change the *relative* response (intensity) compared to the pre‐response condition. Nonetheless, eutrophying systems tend to operate much closer to regulatory thresholds for water quality (e.g., limit concentration of Chl‐*a*), implying that higher absolute responses may already pose significant risks for water security even if the proportion of responses in the different eutrophication levels remain the same.

## CONFLICT OF INTEREST

The authors declare that they have no conflict of interest.

## AUTHOR CONTRIBUTIONS

**Julio A. A. Stelzer:** Conceptualization (lead); Data curation (lead); Formal analysis (lead); Investigation (lead); Methodology (lead); Project administration (equal); Validation (lead); Visualization (lead); Writing – original draft (lead); Writing – review & editing (lead). **Jorrit P. Mesman:** Conceptualization (supporting); Data curation (equal); Formal analysis (equal); Investigation (supporting); Methodology (equal); Project administration (supporting); Validation (equal); Visualization (equal); Writing – original draft (supporting); Writing – review & editing (equal). **Alena S. Gsell:** Conceptualization (supporting); Investigation (supporting); Methodology (supporting); Supervision (supporting); Writing – review & editing (supporting). **Lisette N. de Senerpont Domis:** Conceptualization (equal); Formal analysis (equal); Investigation (equal); Methodology (equal); Project administration (equal); Supervision (lead); Writing – review & editing (supporting). **Petra M. Visser:** Investigation (supporting); Methodology (supporting); Writing – review & editing (supporting). **Rita Adrian:** Formal analysis (supporting); Investigation (supporting); Methodology (supporting); Project administration (supporting); Supervision (supporting); Writing – review & editing (supporting). **Bastiaan W. Ibelings:** Conceptualization (equal); Funding acquisition (lead); Investigation (supporting); Methodology (supporting); Project administration (equal); Supervision (lead); Writing – original draft (supporting); Writing – review & editing (equal).

## Supporting information

Appendix S1

## Data Availability

Data and scripts are available on FigShare digital repository (https://doi.org/10.6084/m9.figshare.16646872).
