# Supplementary material for: Phytoplankton responses to repeated pulse perturbations imposed on a trend of increasing eutrophication
Source: Ecol Evol. 2022 Mar 1;12(3):e8675. doi: 10.1002/ece3.8675 (PMC8888247; doi:10.1002/ece3.8675)
Supplement: Supplementary file 1 — Appendix S1 [file ECE3-12-e8675-s001.doc]

Contents

[Methodology 1](#__RefHeading___Toc94290079)

[Metrics for quantifying response to pulse perturbations 1](#__RefHeading___Toc94290080)

[Statistics 4](#__RefHeading___Toc94290081)

[Long-term effect of the press perturbation (eutrophication) on total biovolume and total chlorophyll-a concentrations 4](#__RefHeading___Toc94290082)

[Biovolume 4](#__RefHeading___Toc94290083)

[Total pigments in function of biovolume and treatment 5](#__RefHeading___Toc94290084)

[Short-term effects of the press perturbation (eutrophication) on the response and recovery from mortality pulse perturbations (chlorophyll-a as a proxy) 6](#__RefHeading___Toc94290085)

[Pre-event condition 6](#__RefHeading___Toc94290086)

[Maximum displacement 7](#__RefHeading___Toc94290087)

[Recovery index 10](#__RefHeading___Toc94290088)

[Resistance index 11](#__RefHeading___Toc94290089)

[Functional structure 12](#__RefHeading___Toc94290090)

[Periphyton formation 13](#__RefHeading___Toc94290091)

[References 14](#__RefHeading___Toc94290092)

# Methodology

## Metrics for quantifying response to pulse perturbations

Table 1. Metrics for quantifying response to pulse perturbations and their formula.

| Metric | Symbol | Formula | Reference |
| --- | --- | --- | --- |
| Pre-event condition | C_o_ | Rolling window of three days with least variance | - |
| Maximum Displacement | D_o_ | 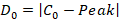 | Orwin and Wardle (2004) |
| Resistance Index | RS | 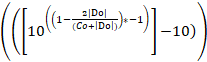*-1 | Modified from Orwin and Wardle (2004) |
| Recovery Index | RC | 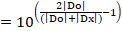  Where D_x_=Post-event condition | Modified from Orwin and Wardle (2004) |

#### Resistance (simulation)


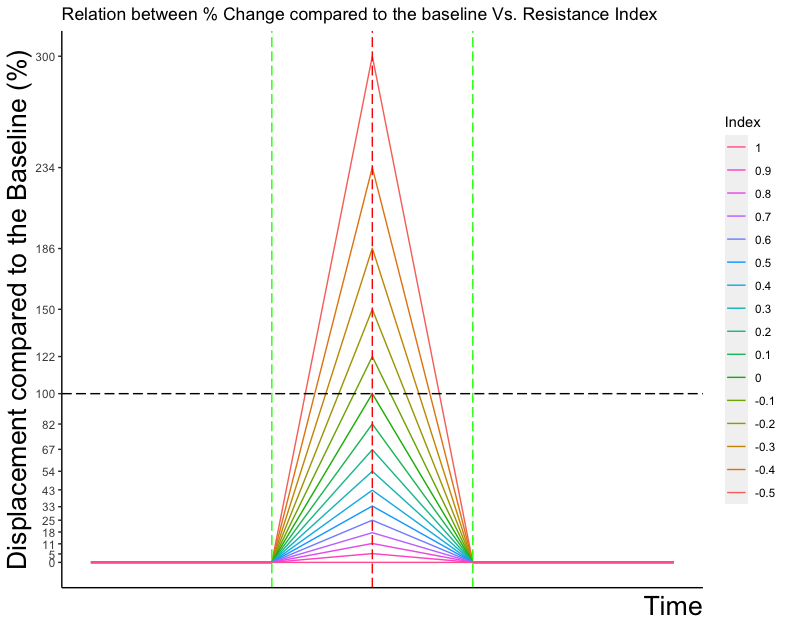


Figure 1. Resistance of simulated data with displacements of different intensities using the index proposed by Orwin and Wardle (2004). RS index = 0 means 100% displacement compared to the baseline


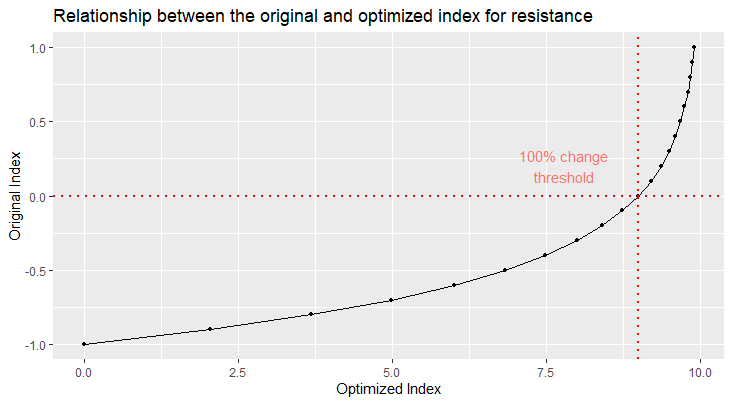


Figure 2. Relationship between the original scaling and optimized scaling used for the resistance index.

#### Recovery (simulation)


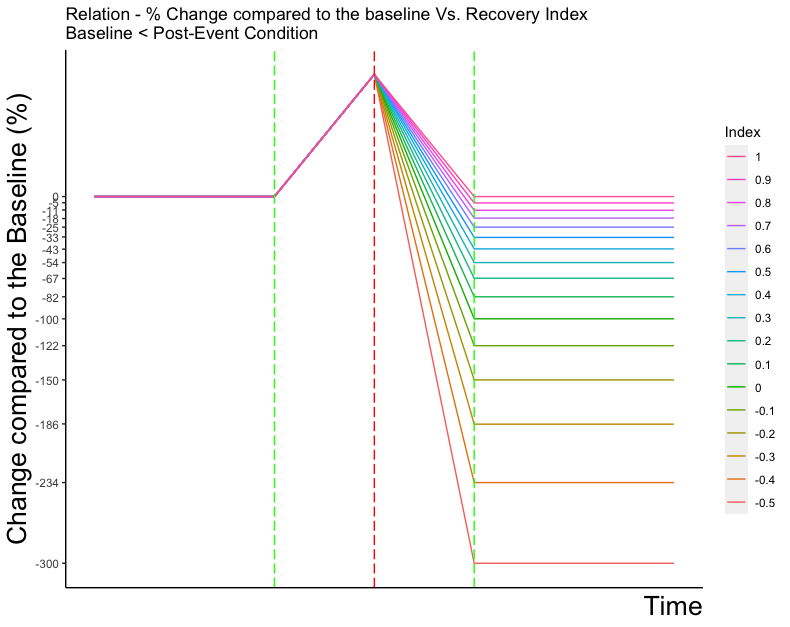

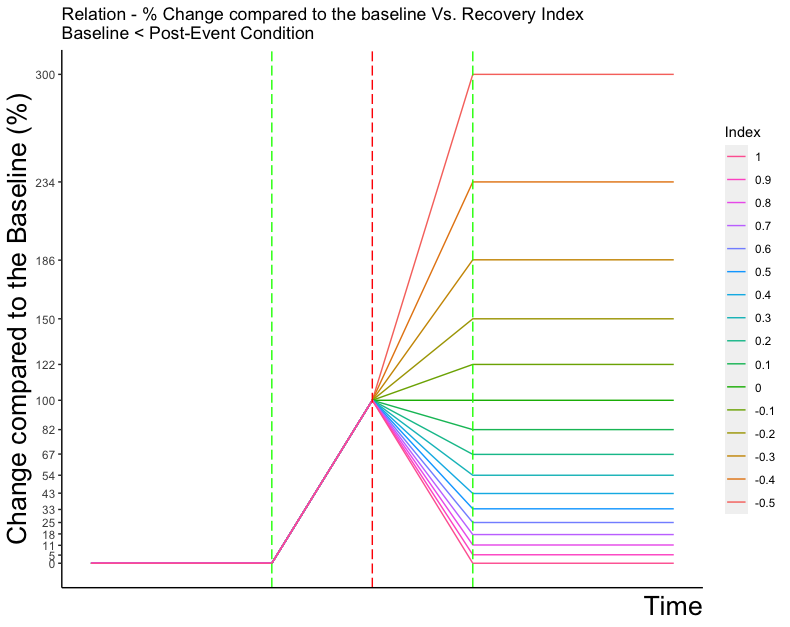


Figure 3. (left) Recovery of simulated data with post-event condition lower than the antecedent baseline. RC index = 0 means 0% recovery. In other words, the system is displaced and stabilizes at the maximum displacement level. Negative RC index values address values of post-event conditions higher than the module of max displacement. (right) Recovery of simulated data with post-event condition higher than the antecedent baseline. RC index = 0 means 0% recovery. In other words, the system is displaced and stabilizes at the maximum displacement level. Negative RC index values address values of post-event conditions higher than the max displacement.


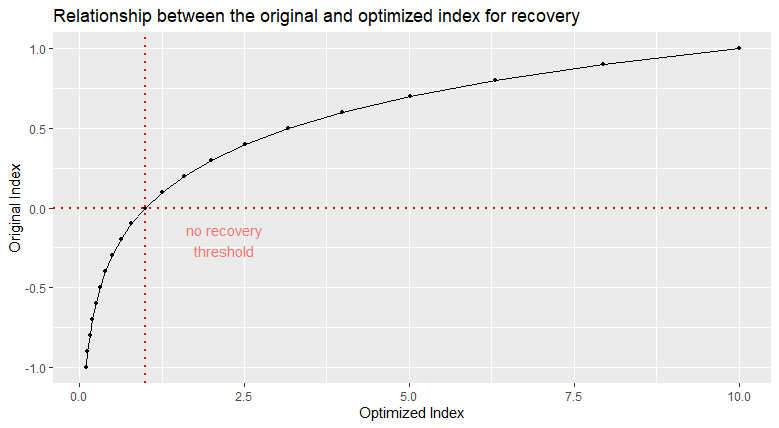


Figure 4 Relationship between the original scaling and optimized scaling used for the recovery index.

# Statistics

## Long-term effect of the press perturbation (eutrophication) on total biovolume and total chlorophyll-a concentrations

### Biovolume

Table 2. Model output from the generalized additive mixed model (GAMM) for total biovolume during the perturbations. Fixed effects: Eutrophication treatment and No. of perturbations; Random effects: Cosm ID (pseudoreplication) and Perturbation Intensity. [m = gam(data=df_biovolume, Log ~ Treatment * Pert + s(ID, bs = 're') + s(Intensity, bs = 're'), method = 'REML').


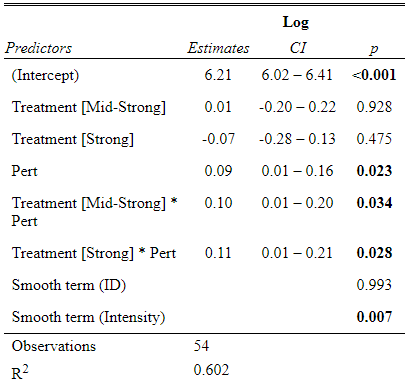


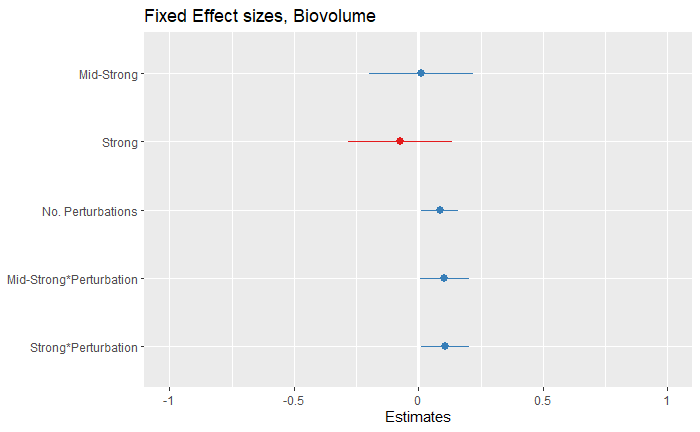


Figure 5. Model estimates for the fixed effects sizes on total biovolume. Random effects are not showed here but can be reproduced using the R.markdown and data available with the manuscript.

### Total pigments in function of biovolume and treatment

Table 3. Model output from the generalized linear mixed model (GLMM) showing the relation of total biovolume and eutrophying treatments to the total concentration of phytoplankton pigments (N=466, R^2^=0.723). [glm(data = df_timeseries, Log_Pig ~ Log_Bio + factor(Treatment))].


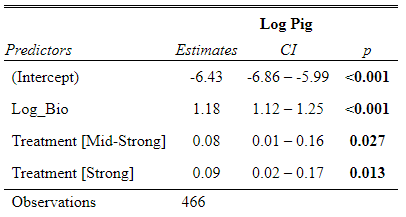


## Short-term effects of the press perturbation (eutrophication) on the response and recovery from mortality pulse perturbations (chlorophyll-a as a proxy)

### Pre-event condition

Table 4. Model output from the linear mixed effect model (LMEM) for pre-event conditions. Fixed effects: Eutrophication treatment and No. of perturbations; Random effects: Cosm ID (pseudoreplication) and Perturbation Intensity. [m = lmer(data=df_baseline, Log ~ Treatment * Pert + (1 | ID) + (1 |Intensity))].


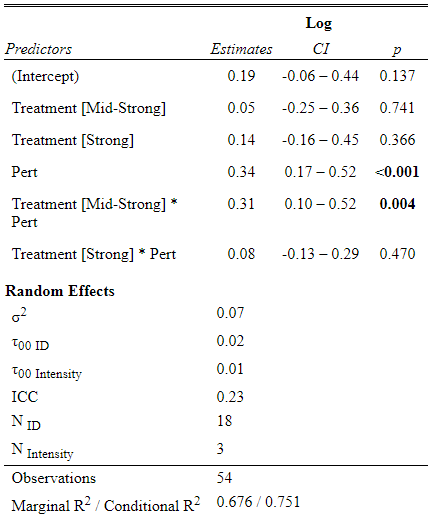


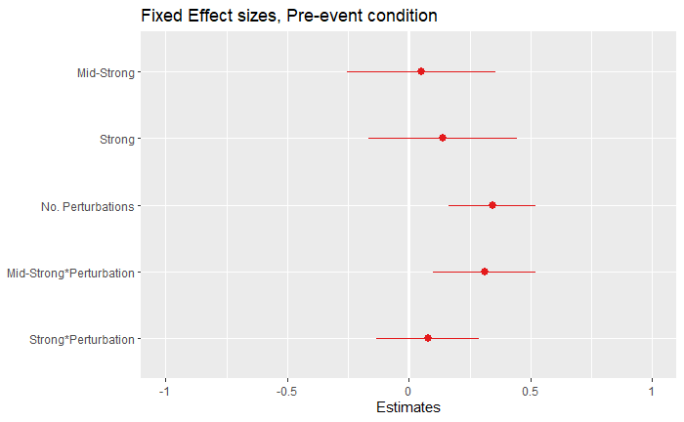

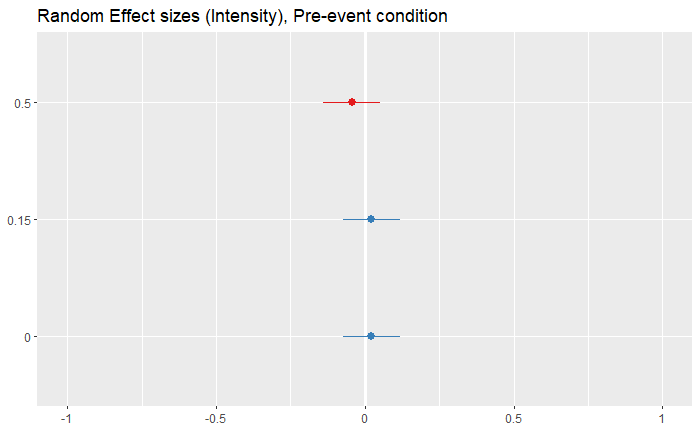


Figure 6. Model estimates for the fixed effects (left) and random effect sizes (right) for pre-event conditions. Random effects for Cosm ID are not showed here but can be reproduced using the R.markdown and data available with the manuscript.

### Maximum displacement

Table 5. Model output from the linear mixed effect model (LMEM) for maximum displacement. Fixed effects: Eutrophication treatment and No. of perturbations; Random effects: Cosm ID (pseudoreplication) and Perturbation Intensity. [m = lmer(data=df_displacement, Response ~ Treatment + Pert + (Pert | ID) + (1 |Intensity ))].


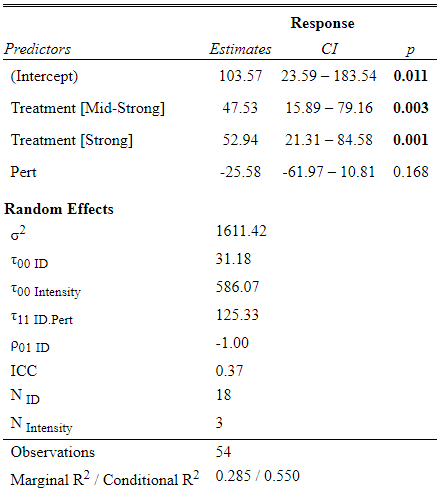


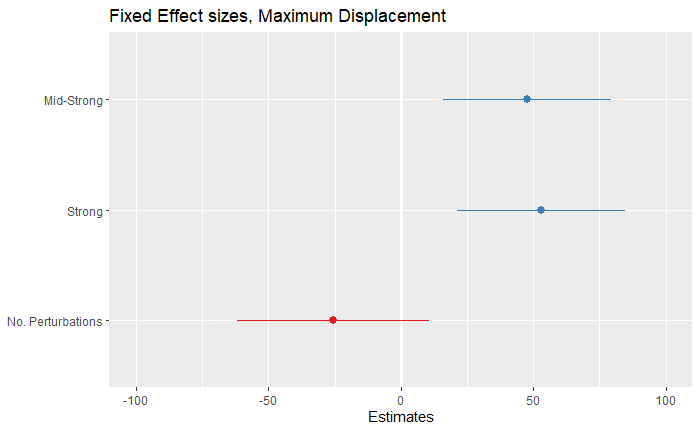

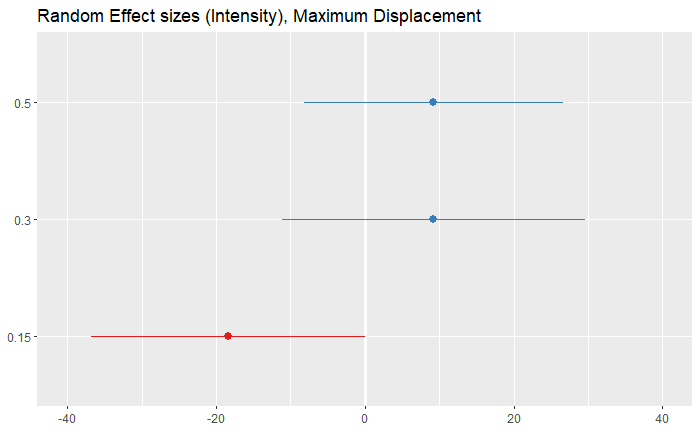


Figure 7. Model estimates for the fixed effects (left) and random effect sizes (right) for maximum displacement. Random effects for Cosm ID are not showed here but can be reproduced using the R.markdown and data available with the manuscript.

#### Importance of intensity

Table 6. Model output from the linear mixed effect model (LMEM) for maximum displacement. Fixed effects: Eutrophication treatment, perturbation intensity and number of perturbations. Random effect: Cosm ID (pseudoreplication). [m = lmer(data=df_displacement, Response ~ Treatment + Intensity + Pert + (1 | ID))].


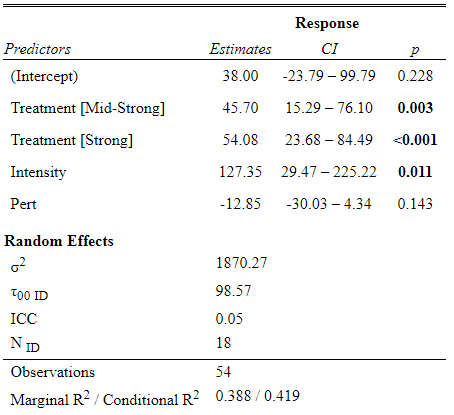


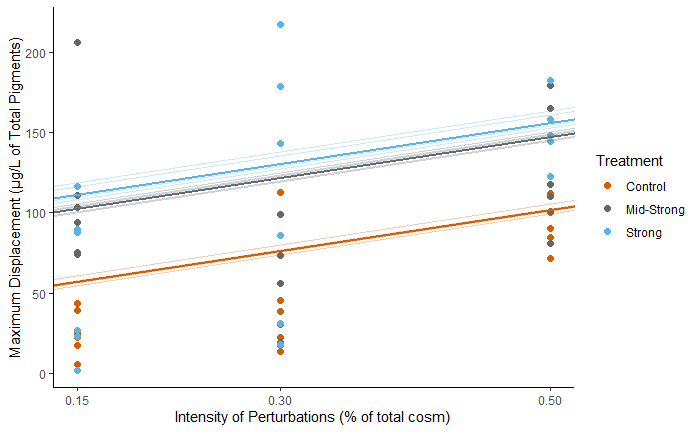


Figure 8. The effect of eutrophication on the maximum displacement after perturbation. Differently from the other assessments in the body of the manuscript, the intensity was also included as a fixed effect here. This is because we were interested in observing the additive effect of intensity and repeated pulse perturbations on the maximum displacement of the system. While the number of sequential perturbations is not apparent in the plot (z-axis), it modifies the intensity and maximum displacement relation. This can be observed in the effect sizes plot in Figure 9. Solid regression line represents the full model estimates, and shaded lines represent the individual cosm estimate without random intercepts. Shaded dots are the empirical data (n=6). Fixed effects: Eutrophication treatment, perturbation intensity, and the number of perturbations. Random effect: Cosm ID (pseudoreplication).


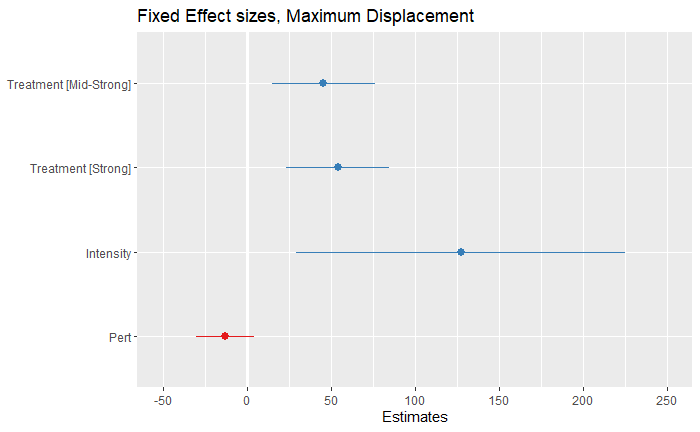


Figure 9. Model estimates for the fixed effects for maximum displacement. Note that the direction of the effect size of the number of perturbations (“Pert”) is in the opposite direction of eutrophication and perturbation intensity. Random effects for Cosm ID are not showed here but can be reproduced using the R.markdown and data available with the manuscript.

### Recovery index

Table 7. Model output from the linear mixed effect model (LMEM) for Recovery Index. Fixed effects: Eutrophication treatment and No. of perturbations; Random effects: Cosm ID (pseudoreplication) and Perturbation Intensity. [m = lmer(data=df_recover, Log ~ Treatment * Pert + (1 | ID) + (1 |Intensity))].


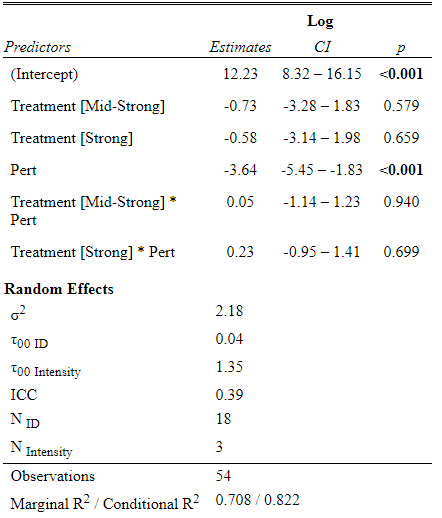


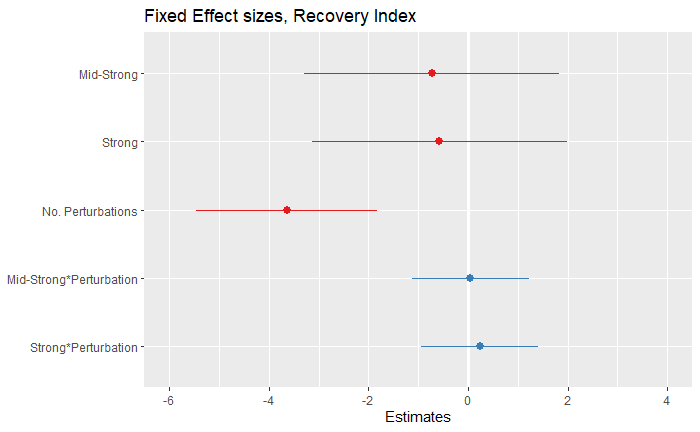

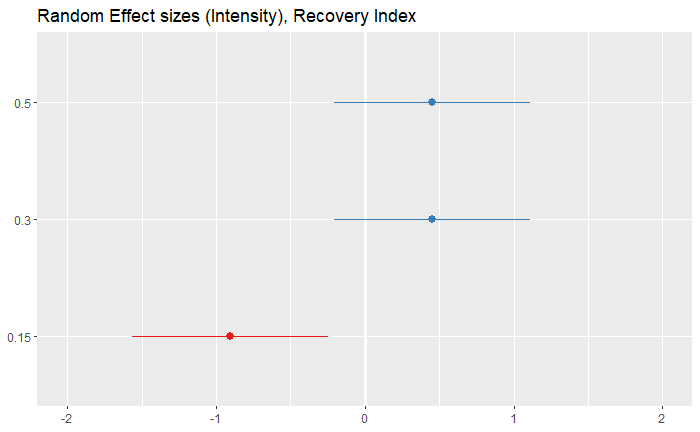


Figure 10. Model estimates for the fixed effects (left) and random effect sizes (right) for recovery. Random effects for Cosm ID are not showed here but can be reproduced using the R.markdown and data available with the manuscript.

### Resistance index

Table 8. Model output from the generalized additive model (GAM) for Resistance Index. Fixed effects: Eutrophication treatment and No. of perturbations; Random effects: Cosm ID (pseudoreplication) and Perturbation Intensity. [m = gam(Log ~ Treatment + Pert + Treatment:Pert + s(Pert, ID, bs = 're') + s(Intensity, bs = 're'), data = df_change, method = 'REML')].


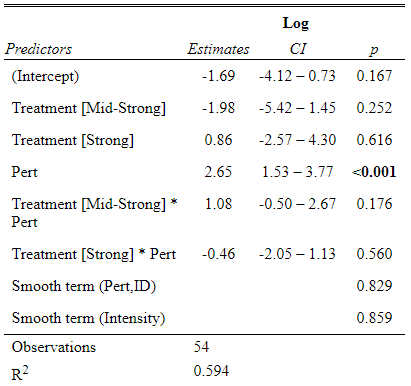


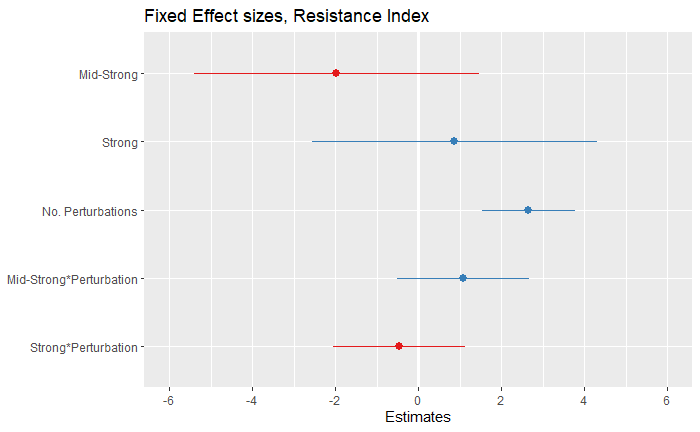


Figure 11. Model estimates for the fixed effects sizes on resistance. Random effects are not showed here but can be reproduced using the R.markdown and data available with the manuscript.

# Functional structure


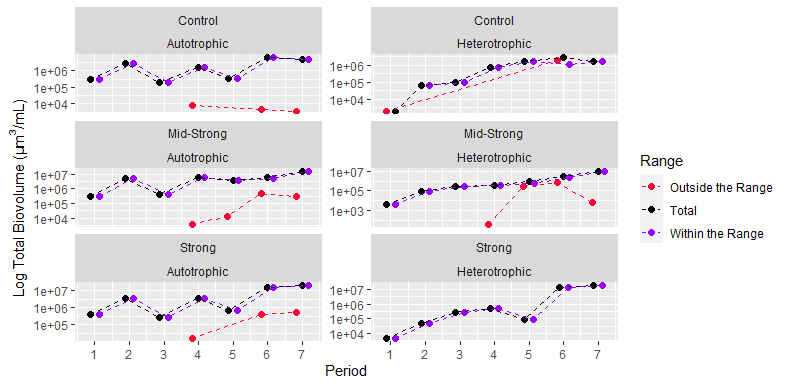


Figure 12. Comparison between the size fractions quantified using the Coulter Counter (purple, fraction 2.93 µm – 60 µm) and when analyzed using microscopy (red, fraction larger than 60 µm). The group outside the range includes filamentous algae, cyclopoids, and one taxon of ciliate larger than 60µm in diameter. The odd numbers in the x-axis represent pre-response conditions (moments of stability before perturbation), and the even numbers represent the peak of phytoplankton response measure with Phyto-PAM™. Data counted using an inverted microscope using pooled samples of the 6 replicates per treatment


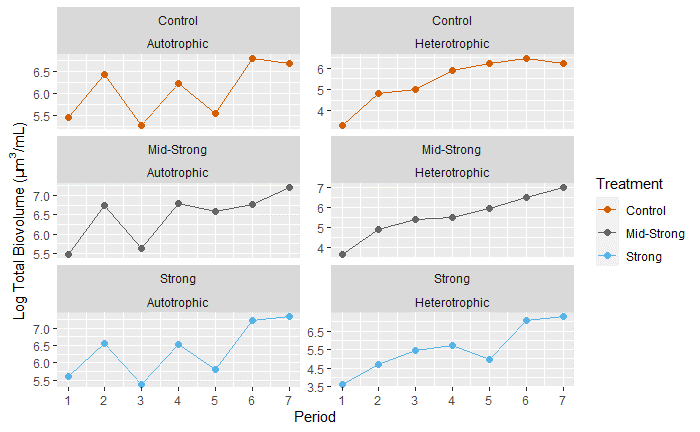


Figure 13. Timeseries of the biovolume concentration of autotrophs Vs. heterotrophs along the three perturbation cycles of the perturbation experiment. The odd numbers in the x-axis represent pre-response conditions (moments of stability before perturbation), and the even numbers represent the peak of phytoplankton response measure with Phyto-PAM™. Data were counted using an inverted microscope using pooled samples of the 6 replicates per treatment. Y-axis log10.


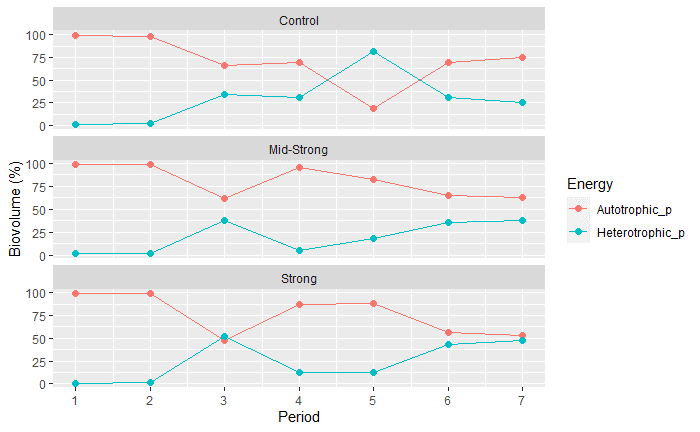


Figure 14. Timeseries of the relative biovolume concentration of autotrophs Vs. heterotrophs along the three perturbation cycles of the perturbation experiment. The odd numbers in the x-axis represent pre-response conditions (moments of stability before perturbation), and the even numbers represent the peak of phytoplankton response measure with Phyto-PAM™. Data were counted using an inverted microscope using pooled samples of the 6 replicates per treatment.

# Periphyton formation

Preparation of periphyton samples:

Squares of 15.76 cm^2^ polycarbonate sheets were cut and placed at the bottom of each mesocosm coupled with the temperature loggers. Sheets were deployed just after the pulse perturbation on the 3^rd^ cycle and removed at the end of the experiment, 17 days later (from day 78 to day 95).

For quantification, each periphyton stripe was removed and placed in a 50mL falcon with 42 ml of NaCl 0.9%. The falcons were vigorously agitated and vortexed at maximum speed at least 30 seconds to lose the periphyton from the polycarbonate sheet.

After, the solution was measured using the Phyto-PAM in the same way as phytoplankton in the experiment, using NaCl 0.9% as blank. For the dry weight, acetate cellulose filters were pre-weighted, 28mL of solution filtered, dried for 24h at 60 degrees, and re-weighted.

Dry weight and Chlorophyll-a concentrations were calculated based on the surface area of the mesocosms (diameter= 26.2 cm, height = 22 cm). The dry weight is presented as absolute mass of periphyton per container (in mg) and Chlorophyll-a as the concentration of Chl-a at the mesocosms surface if diluted in 10L volume (the content of the mesocosm).

Values were compared to the averaged Chl-a concentrations and the cumulative Chl-a concentration in the water column during the same period.

Table 9. Averaged values of periphyton and phytoplankton at the last quarter of the experiment (mean ± sd). Cum_Sum = cumulative sum of Chl-a concentration of the phytoplankton community during the 17 days of periphyton measurement.

|  | Measurement | Control | Mid-Strong | Strong |
| --- | --- | --- | --- | --- |
| Periphyton | Dry Weight (mg) | 53.67 ± 53.67 | 120.35 ± 41.04 | 111.82 ± 24.01 |
|  | Chl-a (µg/L) diluted in 10L mesocosm (Periphyon) | 15.39 ± 8.42 | 34.48 ± 21.32 | 23.74 ± 24.81 |
| Phytoplankton | Chl-a (µg/L)* | 36.62 ± 27.96 | 48.15 ± 38.34 | 79.38 ± 72.44 |
|  | Cum_Sum (µg/L) | 1977.78 | 2622.55 | 4286.47 |

* Note that the high sd values are due to the integration of the timeseries during the perturbation period


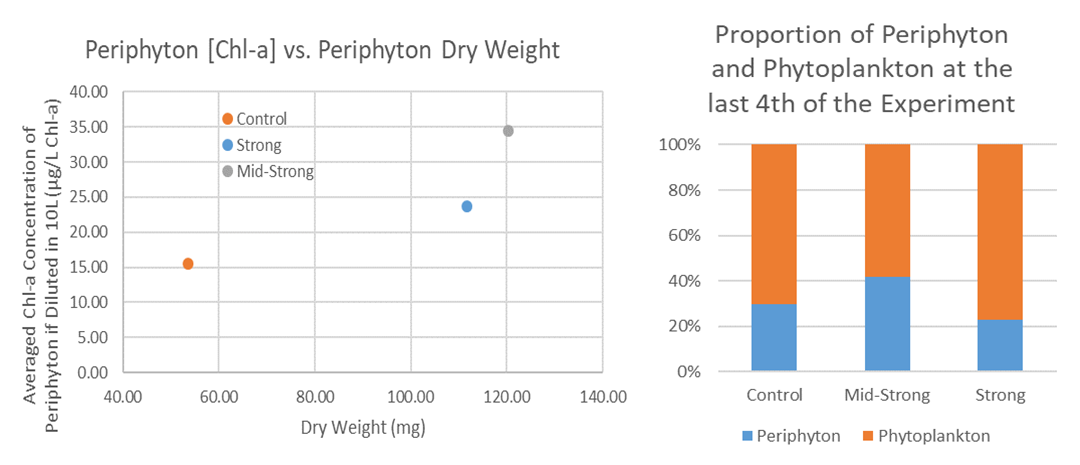


Figure 15. left: Relation between dry weight and chlorophyll-a concentrations in the periphyton community after 17 days of colonization. Note that the increase in dry weight of periphyton is poorly related to the increase in Chl-a concentrations, likely due to heterotrophic microorganisms. Right: Proportion between periphyton and phytoplankton in the last perturbation cycle. Note that the periphyton Chl-a concentration results from 17 days of accumulation, while the phytoplankton Chl-a concentration is the averaged value during the same period.

# References

ORWIN, K. H. & WARDLE, D. A. 2004. New indices for quantifying the resistance and resilience of soil biota to exogenous disturbances*. Soil Biology & Biochemistr*y, **3**6, 1907-1912.
